# Evaluating the Effect of the JUUL2 System With 5 Flavors on Cigarette Smoking and Tobacco Product Use Behaviors Among Adults Who Smoke Cigarettes: 6-Week Actual Use Study

**DOI:** 10.2196/60620

**Published:** 2025-03-26

**Authors:** Nicholas I Goldenson, Saul Shiffman, Mark A Sembower, Arielle Selya, Steve Pype, Ryan A Black

**Affiliations:** 1 Juul Labs, Inc Washington, DC United States; 2 PinneyAssociates, Inc Pittsburgh, PA United States

**Keywords:** cigarette, smoking, electronic nicotine delivery system, switching, actual use study, real world

## Abstract

**Background:**

Adults who switch completely from smoking cigarettes to using electronic nicotine delivery systems (ENDS) substantially reduce their exposure to toxicants and carcinogens that are associated with smoking-related diseases.

**Objective:**

This 6-week actual use study—a prospective uncontrolled real-world study designed to evaluate quasi-naturalistic product use—aimed to assess switching behavior among US adults who smoked cigarettes and were provided with JUUL2 ENDS products.

**Methods:**

US adults who smoked cigarettes every day but were predominantly not ready to quit (N=1160; mean age 39.42, SD 11.03 years; 641/1160, 55.26% female participants; 667/1160, 57.5% non-Hispanic White; mean cigarettes per day 14.11, SD 8.96; only 1% [11/1160] planning to stop smoking within 30 days; and 481/1160, 41.47% dual users) were recruited to use JUUL2 ENDS products (18 mg/mL nicotine) in 1 of 5 flavors in real-world environments for 6 weeks. Participants who expressed sufficient interest in using JUUL2 products were enrolled at 24 different consumer research sites across the United States into one of the two following study arms: (1) traditional flavors (Virginia Tobacco and Polar Menthol, 10 sites); or (2) complex flavors (Autumn Tobacco, Summer Menthol, and Ruby Menthol, 14 sites). No instructions regarding JUUL2 product use or cigarette smoking were provided. After a 1-week trial period, participants were provided with their preferred flavor for 6 weeks of ad libitum use (10 pods per week). In total, 6 weekly web-based surveys were used to assess switching (smoking abstinence) and smoking reduction; dependence and respiratory symptoms were assessed at baseline and week 6.

**Results:**

Across the 5 flavor groups at week 6, the rates of complete past-7-day switching away from cigarettes ranged from 38.2% (79/207) to 47.3% (95/201), and 24.3% (55/226) to 33.9% (74/218) of participants reported complete past-30-day switching. Participants who used the 3 menthol-flavored (vs 2 tobacco-flavored) JUUL2 products had significantly higher rates of past-30-day switching at week 6 (odds ratio 1.36, 95% CI 1.04-1.78). Compared to their baseline values when they were smoking, the past-30-day switchers at week 6 had significantly reduced their dependence (mean differences in dependence, cigarettes – JUUL2: 0.57-0.99; *P*<.001) and self-reported frequency of respiratory symptoms (*P*<.05). Among participants who continued to smoke at week 6, 50.9% (59/116) to 62.9% (73/116) reduced their daily cigarette consumption by at least 50% from baseline.

**Conclusions:**

Adoption of JUUL2 ENDS products can likely help substantial proportions of US adults who smoke to switch completely away from cigarettes or meaningfully reduce their cigarette consumption, thereby reducing their dependence on tobacco products and improving their respiratory symptoms.

## Introduction

### Background

Evidence demonstrates that adults who switch completely from smoking cigarettes to using electronic nicotine delivery systems (ENDS) substantially reduce their exposure to toxicants and carcinogens that are associated with smoking-related diseases [1-4]. Accordingly, the primary public health benefit of ENDS is derived from facilitating switching behavior among adults who smoke.

For ENDS products that are currently marketed in the United States, observational studies and randomized trials have assessed switching behavior among adults who smoke [5-9]. However, for premarket ENDS products (ie, those not currently marketed in the United States), evaluating naturalistic product use behaviors and transitions over time in real-world settings requires providing the premarket product to adults who smoke cigarettes. Actual use studies—prospective uncontrolled real-world studies designed to evaluate quasi-naturalistic product use—are the primary method for assessing the use of premarket tobacco products under ecologically valid conditions. Actual use studies were initially developed for pharmaceuticals to determine whether prescription drugs could be switched to over-the-counter sale by assessing use and safety of the drug in real-world settings designed to mimic over-the-counter use [10,11].

In recent years actual use studies have evaluated use of tobacco products, including oral nicotine pouches [12,13] and heated tobacco (heat-not-burn) products [14,15], among adults who smoke to prospectively assess changes in cigarette smoking following quasi-naturalistic use of the new tobacco products in real-world settings. In contrast to randomized cessation trials, actual use studies do not require participants to have active plans to quit smoking to qualify, do not set explicit goals for product use or smoking, and do not provide instructions, encouragement, or behavioral support to quit. Thus, actual use studies are particularly well-suited to evaluate the ability of ENDS to facilitate switching behavior (smoking abstinence) following periods of unguided self-determined use.

The JUUL2 system is a next-generation pod-based ENDS product that is designed to facilitate high levels of complete switching away from cigarettes among adults who smoke cigarettes. Pharmacokinetic studies show that JUUL2 18 mg/mL (1.5% nicotine by weight) products can deliver similar levels of nicotine as ENDS products with higher nicotine concentrations due to increased aerosol production [16], and behavioral pharmacology data demonstrate that Polar Menthol–flavored 18 mg/mL JUUL2 pods are rated as significantly more satisfying than menthol-flavored pod-based ENDS with 5% nicotine concentrations [17]. The JUUL2 system is currently marketed in the United Kingdom; however, there is a lack of real-world data on the switching potential of these JUUL2 products among US adults who smoke.

### Objectives

The primary aim of this actual use study was to evaluate complete switching away from cigarettes (ie, past-7-day and past-30-day smoking abstinence) among US adults who smoked cigarettes every day and were provided with JUUL2 products in their preferred flavor for 6 weeks. Secondary aims of the study were to assess: (1) changes in dependence and respiratory symptoms among participants who switched completely away from smoking at week 6; (2) changes in cigarette consumption among participants who continued smoking at week 6; (3) patterns of JUUL2 product use and subjective responses to JUUL2 products; and (4) factors associated with switching behavior, including JUUL2 study product flavor (menthol vs tobacco), cigarette flavor of usual brand (menthol vs nonmenthol), and baseline ENDS use (dual use along with cigarette smoking).

## Methods

### Design

The study was designed as an actual use study—a prospective real-world study intended to simulate quasi-naturalistic product use—in which US adults who smoked cigarettes were provided with JUUL2 study products in participant-selected flavors for 6 weeks of ad libitum use in their natural environment. All potential participants first completed an initial prescreening survey (in person or via telephone) and then the formal recruitment screening on the web. The study had 2 distinct arms, implemented at separate research sites with different geographic catchment areas that tested 1 of the 2 following flavor series: (1) traditional flavors (Virginia Tobacco and Polar Menthol), which correspond to ENDS flavors that have been authorized for marketing by US Food and Drug Administration (FDA; 10 sites); or (2) complex flavors (Autumn Tobacco, Summer Menthol, and Ruby Menthol), which represent adult-oriented flavor mixtures that have not yet received market authorization from FDA (14 sites). The 2 arms were conducted at distinct sites and did not share participant pools to evaluate traditional and complex flavors independently. The decision to separate the traditional and complex flavors was an a priori design based on differences in the composition of traditional and complex flavors (see Study Products section), with implied different regulatory positions (ie, FDA has authorized tobacco- and menthol-flavored ENDS, but has not yet authorized nontobacco or nonmenthol flavors or blends), and to avoid the complexity of each participant and site testing all 5 flavors. Study design and procedures for the 2 arms were identical, and the geographic and demographic position of the research sites were roughly parallel, but sites recruited participants from their local catchment area, thus there was no overlap in potential participants across study sites. Importantly, participants were not assigned to sites or arm by the study (randomly or otherwise) but were recruited from each site’s distinct geographic catchment area. The study sponsor did not play any role in the recruitment of participants and individuals were primarily recruited from existing databases maintained by the study sites or in person (not sponsor databases).

Eligible participants first completed a 1-week trial period to determine which flavor they would exclusively use for the 6-week actual use period. During the 6-week actual use period, participants completed weekly web-based survey assessments (Multimedia Appendix 1).

At prescreening, participants were informed that the research study involved using new electronic cigarette products for 7 weeks and that participation in the study did not require stopping smoking cigarettes—the study was not framed or described as a study of behavior change or smoking cessation. During the trial week and 6-week actual use period, no goals or instructions were provided regarding cigarette smoking; participants could smoke ad libitum throughout the study, or reduce or forego smoking, at their discretion. Similarly, no instructions (beyond the user guide included in the product package) were provided regarding use of the JUUL2 study products with respect to amount or persistence of use. The only directions were those that come with marketed products, describing how to use the product and how to charge the device.

### Participants

The study sample included healthy non–treatment-seeking English-speaking US adults (aged 22 to 65 years) who smoked cigarettes every day and lived close to one of the 24 shopping-mall–based study sites distributed across 15 states (Multimedia Appendix 2). Eligibility criteria were as follows: (1) smoked ≥100 combustible tobacco cigarettes in their lifetime and smoked cigarettes for ≥12 months before screening; (2) smoked cigarettes every day and smoked an average of ≥5 cigarettes per day; (3) interested in trying and using JUUL ENDS products; (4) access to a computer or laptop, smartphone, or tablet with internet access; (5) provided consent for participation and acknowledged willingness and ability to comply with all study requirements. Interest in stopping smoking was not an eligibility criterion.

Exclusion criteria included the following: (1) use of any nicotine replacement therapy (eg, patch and gum) or prescription smoking cessation medications (eg, varenicline and bupropion) within 30 days before screening; (2) diagnosis of a clinically significant medical (eg, asthma, chronic obstructive pulmonary disease, heart disease, high blood pressure, and cancer) or psychiatric disease; (3) was pregnant, nursing, or intended to become pregnant at any time through the end of study; (4) participated in a research study about tobacco products or ENDS within the past 30 days; (5) was a current or former employee or related to a current or former employee of the tobacco or ENDS industry or any vendor associated with conduct of the study; and (6) operated or lived with family member who operated any home childcare or health care services. There were no eligibility criteria regarding prior use of ENDS; participants could be ENDS naive, former ENDS users, or current dual users. Recruitment aimed to enroll a diverse sample with respect to sociodemographic and tobacco product use characteristics, according to the following soft quotas: (1) ≤60% of male or female participants; (2) at least 35% of participants who identified as non-Hispanic White; (3) ≥50% participants aged <45 years; (4) ≥40% of participants who smoked mentholated cigarettes; and (5) ≥50% of participants who had ever used ENDS. Individuals were primarily recruited from existing databases maintained by the study sites and the study sponsor did not play any role in recruitment of participants.

### Ethical Considerations

Participants provided written informed consent. All surveys were completed remotely on the web. Participants were compensated US $20 for completing the baseline assessment, US $15 for completing the product trial assessment, and US $25 for completing each weekly assessment. Additional compensation was provided for completing all study assessments and returning study products, with maximum total compensation of US $450. The Advarra Institutional Review Board approved the study protocol (Pro00068019).

### Procedure

Following enrollment, participants completed the baseline survey on the web and visited their study site, where they were provided with a JUUL2 device, charging dock, and 2 pods of each of the flavors in their respective study arm (2 traditional flavors or 3 complex flavors) for a 1-week product trial period. Participants were instructed to use each flavor for at least one day, enough to form an opinion of the product; no other instructions were provided. After the trial period, participants completed a brief web-based survey in which they rated their interest in each of the trialed JUUL2 pod flavors. These responses were used to determine if participants qualified for the 6-week actual use period and which flavor they would use (see Interest in Using and Purchasing JUUL2 Products subsection of Measures).

At the start of week 1, participants received an additional JUUL2 device. Every 2 weeks (at the start of week 1, week 3, and week 5) participants were provided with 20 pods for use during the upcoming 2 weeks (10 pods per week, 60 pods total). Participants were invited by email to complete assessments at the end of each of the study weeks and received compensation upon completion of each weekly assessment. Participants had 3 days from the initial invitation to complete the weekly surveys and they received multiple reminder emails prompting them to complete the assessment (surveys not completed within this window were considered missing).

Participants were instructed in the informed consent form and by study staff to inform study staff of any adverse events (AEs) and intercurrent illnesses experienced during the study. In addition, a specific inquiry regarding AEs (ie, if participants had experienced any changes to their health [yes or no]) was made at the product trial (week 0), week 3, and week 6 surveys; at the week 1, 2, 4, and 5, surveys participants were prompted to report any changes to their health to the study staff. Reported health events were assessed and classified via clinical interviews by trained medical personnel.

### Study Products

The JUUL2 system is closed-system pod-based ENDS that is inhalation-actuated and does not have any user-modifiable settings, controls, or buttons. Preclinical studies demonstrate that the aerosol produced by the JUUL2 system contains substantially lower levels of harmful and potentially harmful constituents than cigarette smoke [18]. The 5 JUUL2 pod flavors evaluated in this study are all currently marketed in the United Kingdom. Virginia Tobacco and Polar Menthol have traditional tobacco and menthol flavors, respectively. The 3 complex flavors (Autumn Tobacco, Summer Menthol, and Ruby Menthol) consist of a tobacco or menthol base, respectively, with top notes of fruit: apple for Autumn Tobacco, tropical fruit for Summer Menthol, and berry for Ruby Menthol.

### Measures

#### Participants Sociodemographic and Tobacco Use Characteristics

Sociodemographic and tobacco or nicotine product use characteristics were assessed in the recruitment screener and baseline assessment with items and measures adapted from national surveys, with some modified for relevance.

#### Interest in Using and Purchasing JUUL2 Products

At the end of the trial week, participants were asked 2 questions about each of the JUUL2 products they used as follows: (1) “How interested are you in using the JUUL2 study product for the next 6 weeks?” (5-point response scale, from 1 [“not at all interested”] to 5 [“extremely interested”]) and (2) “If the JUUL2 study products you tried this week were available for purchase, would you buy one or more of the products?” (4-point response scale, from 1 [“definitely would not buy”] to 4 [“definitely would buy”]). Only participants who responded 3 or higher to both items for at least one flavor continued to the 6-week actual use period. This was intended to mirror real-world self-selection for product use, as individuals who were not interested in using or purchasing a specific flavor of a consumer product would be unlikely to purchase and adopt it. Participants were provided with the single flavor they expressed the greatest interest in using for the 6-week period; those who expressed exactly equivalent interest in multiple flavors were assigned to the flavor group in their respective study arm that was most in need of enrollees, which helped balance the sample size of flavor groups.

#### Cigarette Smoking

At each weekly survey, participants were asked if they had smoked cigarettes (even one puff) in the past 7 days (yes or no). At the week 6, survey participants were asked if they smoked in the past 30 days: “no” responses were operationalized as switching. Participants who reported smoking in the past 7 days at any survey between weeks 3 and 6 (ie, within 30 days of week 6) were coded as past-30-day smokers, even if they did not complete the week 6 survey or reported past-30-day abstinence at week 6.

Participants who reported smoking in the past 7 days also reported frequency and intensity of cigarette smoking during that period—daily cigarette consumption was calculated as cigarettes smoked per day in the past 7 days, including nonsmoking days (ie, product of the past-7-day frequency and daily intensity).

#### Dependence on Cigarettes and JUUL2 Products

Dependence on cigarettes was assessed at baseline and dependence on JUUL2 products was assessed at week 6 among participants who had not smoked in the past 30 days with the Tobacco Dependence Index (range 1-5, with higher scores indicating greater levels of dependence), a measure psychometrically validated in the Population Assessment of Tobacco and Health study for cross-product comparisons [19,20].

#### Respiratory Symptoms

Frequency of respiratory symptoms was assessed at baseline, when all participants smoked cigarettes every day, and at week 6 among the past-30-day switchers using the Respiratory Symptoms Experience Scale (range 1-5, with higher scores indicating greater frequency of respiratory symptoms), which was developed and validated for adults who smoke cigarettes but may not have clinical respiratory disease [21].

#### Patterns of JUUL2 Product Use

At each weekly survey, participants who reported using JUUL2 products in the past 7 days reported the number of days they used JUUL2 products and completed 2 items that assessed intensity of use as follows: (1) number of use episodes per day and (2) number of puffs per day.

#### Subjective Responses to JUUL2 Products

Subjective responses to JUUL2 products were assessed at week 6 with the modified Product Evaluation Scale [22].

### Statistical Analysis

The study was prospectively powered for its primary aim, to evaluate rates of the past-30-day switching using the Clopper-Pearson exact method [23] with an estimated expected switch rate of 20% (based on switch rates observed in a prior observational study of US adults who purchased JUUL products), achieving a CI of 5%. Power analyses were not conducted for the secondary analyses.

The rate of past-7-day switching was calculated at each weekly survey within each of the 5 flavor groups; the rate of past-30-day switching was also evaluated at the week 6 Survey. A logistic regression model tested the association of JUUL2 flavor group and past-30-day switching at week 6, with Virginia Tobacco as the reference group; separate models contrasted the 2 combined tobacco flavors (Virginia and Autumn Tobacco) to the 3 combined menthol flavors (polar, summer and Ruby Menthol), and the 3 individual menthol flavors, respectively, to Virginia Tobacco. A repeated-measure logistic regression model included the past-7-day switching averaged across all 6 weeks as the dependent variable and JUUL2 flavor group, menthol cigarette preference (mentholated vs nonmentholated), and time (week) since baseline (coded as continuous, range 1-6) as simultaneous regressors—all 2-way interaction terms and the 3-way JUUL2 flavor × cigarette flavor × time interaction term were also included.

A separate repeated-measure logistic regression model was used to assess the association of baseline ENDS use (past 30 days vs not past 30 days) and the past-7-day switching. In addition, mixed effects models evaluated linear changes over time in metrics of daily JUUL2 product use across the 6-week period.

Paired *t* tests were used to assess within-person changes in levels of dependence from baseline to week 6 (from cigarettes to JUUL2 products) among participants who reported no past-30-day smoking at week 6; dual users were not included in this analysis as they continued to smoke cigarettes and their dependence on JUUL2 could not be isolated. Changes in respiratory symptoms from baseline to week 6 were similarly assessed among complete 30-day switchers to assess whether switching affected their respiratory symptoms. Among participants who reported past-7-day smoking at week 6, changes in daily cigarette consumption (cigarettes per day on smoking days) from baseline were also tested with paired *t* tests. The proportion of participants reporting reductions of ≥50% was also calculated.

The primary analyses of switching used all observed (nonmissing) data without imputing smoking status to missed observations. This approach is typical for observational studies [24], in contrast to smoking cessation treatment trials, where there is concern that participants might avoid reporting smoking to avoid admitting to “failure” in an agreed behavioral goal [25]. To assess the potential for missingness to introduce bias, analyses were performed to compare participant baseline characteristics between participants who completed all 6 weekly surveys and those who missed any follow-ups. As a sensitivity analysis, the primary switching analyses were repeated while imputing smoking to all missing observations (intent-to-treat).

In addition, among participants who smoked mentholated cigarettes, differences in sociodemographic and tobacco use characteristics were compared between those who selected tobacco-flavored and menthol-flavored JUUL2 products.

Data were analyzed using SAS version 9.4 (SAS Institute Inc) with alpha level set to 0.05; no adjustments were made for multiple testing.

## Results

### Participant Accrual and Disposition

The enrollment flow in the traditional and complex flavor arms is provided in Multimedia Appendices 3 and 4, respectively. In the traditional flavors arm, 1874 adults were invited to complete the prescreener; 72.68% (1362/1874) completed it and were sent the eligibility screener; and 54.99% (749/1362) subsequently completed the recruitment screener and met all eligibility criteria. Of the eligible participants, 667 (89.1%) provided informed consent and were sent the baseline assessment (Multimedia Appendix 3). Approximately 99% (661/667) of those who started the baseline assessment completed it and were invited to participate in the trial week, 78.8% (521/661) subsequently completed the trial week assessment and were eligible to continue into the 6-week actual use period; 87% (575/661) completed the trial week survey; and 8.2% (54/661) were ineligible to continue into the 6-week actual use period based on expressing insufficient interest in using the study products.

In the complex flavors arm 2625 adults were invited to complete the prescreener; 71.7% (1882/2625) completed it and were sent the eligibility screener and 56.7% (1067/1882) subsequently completed the recruitment screener and met all eligibility criteria. Of the eligible participants, 942 (88.3%) provided informed consent and were sent the baseline assessment (Multimedia Appendix 4). Approximately 98% (927/942) of those who started the baseline assessment completed it and were invited to participate in the trial week, 79.9% (741/927) subsequently completed the trial week assessment and were eligible to continue into the 6-week actual use period; 13.6% (126/927) did not complete the trial week survey; and 6.5% (60/927) were ineligible to continue into the 6-week actual use period because they did not express sufficient interest in using the study products.

At screening, 61 (1.9%) individuals were excluded due to lack of interest in trying JUUL products. At the end of the trial week, the mean ratings of interest in purchasing JUUL2 products ranged from 3.37 to 3.44 on the 4-point response scale; ratings of interest in individual JUUL2 flavors ranged from 3.65 (Virginia Tobacco) to 4.12 (Polar Menthol) on the 5-point response scale (Multimedia Appendix 5). In the traditional flavors arm, 42.2% (242/574) of the participants expressed equivalent interest in both flavors (Virginia Tobacco and Polar Menthol), and in the complex flavors arm, 29.3% (232/792) expressed equivalent interest in all 3 flavors—27.3% (216/792) rated 2 of the 3 flavors equally and 43.4% (344/792) expressed a unique preference for a single flavor. There was no significant difference in the proportion of participants in the traditional (54/661, 8.2%) and complex (60/927, 6.5%) flavors arms that did not continue in the study due to lack of interest in using JUUL2 products at the end of the trial week (χ^2^_1_=1.67, *P*=.20). In the traditional flavors arm, 20.3% (106/521) of the participants met the threshold for inclusion for only one flavor. In the complex flavors arm, 30.6% (227/741) qualified for 1 or 2 flavors (ie, did not meet threshold for at least one flavor) and 8.8% (65/741) qualified for only one flavor.

The analytic sample consisted of 1160 total participants, roughly evenly divided into the 5 flavor groups: 242 participants in the Virginia Tobacco group, 239 in the Polar Menthol group, 219 in the Autumn Tobacco group, 236 in the Summer Menthol group and 224 in the Ruby Menthol group (Multimedia Appendices 3 and 4).

### Sample Characteristics

Characteristics of the sample are reported for all participants (Table 1) and separately within each of the 5 individual flavor groups (Multimedia Appendix 6). Baseline characteristics were generally similar between JUUL2 flavor groups, although larger proportions of participants who selected menthol-flavored JUUL2 products smoked mentholated cigarettes (Multimedia Appendix 6). The participants (mean age 39.42, SD 11.03 years) were majority female (641/1160, 55.26%) and primarily self-identified as non-Hispanic White (667/1160, 57.5%); 19.22% (223/1160) self-identified as non-Hispanic Black; and 16.47% (191/1160) self-identified as of Hispanic ethnicity (Table 1). Over half (632/1160, 54.48%) of them reported annual household income less than US $50,000 and 39.57% (459/1160) did not complete education beyond high school. On average, the participants reported smoking for 16.20 (SD 9.87) years; currently smoked 14.11 (SD 8.96) cigarettes per day and reported high levels of dependence on cigarettes (Tobacco Dependence Index-Cigarettes, mean 3.65, SD 0.81); 73.3% (850/1159) smoked mentholated cigarettes; and only 0.95% (11/1160) planned to quit smoking in the next 30 days at baseline (832/1160, 71.72% planned to never quit smoking).

Nearly three-quarters (825/1160, 71.12%) of the participants had ever used ENDS and, of those, 58.3% (481/825) had used ENDS in the 30 days preceding baseline (ie, were dual users). Dual users, on average, used ENDS 15.25 (SD 9.82) days out of the past 30 days. Among current ENDS users, the use of menthol-flavored products (183/481, 38%) was more common than use of tobacco-flavored ENDS (50/481, 10.4%). JUUL was selected as the primary ENDS brand by 34.5% (166/481) of those currently using ENDS.

**Table 1:** Sociodemographic and tobacco use characteristics of the overall sample (N=1160).

Sample characteristics			Values
**JUUL2 flavor selection, n (%)**			
	Virginia Tobacco		242 (20.86)
	Polar Menthol		239 (20.6)
	Autumn Tobacco		219 (18.88)
	Ruby Menthol		224 (19.31)
	Summer Menthol		236 (20.34)
**Sociodemographic characteristics**			
	Age (y), mean (SD)		39.42 (11.03)
	**Sex, n (%)**		
		Male	517 (44.57)
		Female	641 (55.26)
		Other	1 (0.09)
		Prefer not to answer	1 (0.09)
	**Race or ethnicity, n (%)**		
		Hispanic	191 (16.47)
		Non-Hispanic Black	223 (19.22)
		Non-Hispanic other race^a^	66 (5.69)
		Non-Hispanic White	667 (57.5)
		Unknown	13 (1.12)
	**Marital status, n (%)**		
		Married	369 (31.81)
		Living with partner	222 (19.14)
		Divorced, separated, or widowed	167 (14.4)
		Never married	379 (32.67)
		Prefer not to say	23 (1.98)
	**Annual household income, n (%)**		
		<US $50,000	632 (54.48)
		US $50,000-US $99,999	408 (35.17)
		≥US $100,000	120 (10.34)
	**Highest level of education, n (%)**		
		High school graduate or less	459 (39.57)
		Some college or trade school	422 (36.38)
		College graduate or more education	279 (24.05)
	**Employment status, n (%)**		
		Full-time	754 (65)
		Part-time	145 (12.5)
		Other	261 (22.5)
	**Census region, n (%)**		
		Northeast	54 (4.66)
		Midwest	356 (30.69)
		South	606 (52.24)
		West	144 (12.41)
**Cigarette smoking characteristics**			
	Smoke mentholated cigarettes, n (%)		850 (73.34)
	Number of cigarettes smoked per smoking day, mean (SD)		14.11 (8.96)
	Duration of smoking (y), mean (SD)		16.20 (9.87)
	Age started smoking (y), mean (SD)		19.33 (6.27)
	Cigarette dependence^b^, mean (SD)		3.65 (0.81)
	Plan to quit smoking in next 30 days, n (%)		11 (0.94)
	Ever plan to quit smoking, n (%)		328 (28.28)
**ENDS^c^ use characteristics**			
	Ever used ENDS, n (%)		825 (71.12)
	Age first used ENDS (y), mean (SD)		31.31 (11.4)
	Ever used ENDS fairly regularly, n (%)		529 (64.12)
	Used ENDS in past 30 days, n (%)		481 (58.3)
	Number of days used ENDS in past 30 days, mean (SD)		15.25 (9.82)
	Number of times used ENDS per use day, median (IQR)		9 (5-18)
	ENDS dependence^b^, mean (SD)		3.03 (0.97)
	**Primary ENDS flavor^d^, n (%)**		
		Tobacco	50 (10.4)
		Menthol	183 (38.1)
		Mint	55 (11.4)
		Fruit	152 (31.6)
		Dessert or candy	33 (6.9)
		Spice or clove	2 (0.4)
		Some other flavor	6 (1.3)
	**Primary ENDS device type^d^, n (%)**		
		Pod based	168 (34.9)
		Disposable	229 (47.6)
		Tank	56 (11.6)
		Mod	8 (1.7)
	**Primary ENDS brand^d^, n (%)**		
		JUUL	166 (34.5)
		Vuse	63 (13.1)
		Blu	51 (10.6)
		NJOY	29 (6)
		Puff bar	67 (13.9)
		Other	105 (21.8)

^a^Non-Hispanic other race includes Asian, Pacific Islander or Native Hawaiian, American Indian or Alaska Native, and “another race not listed.”

^b^Tobacco Dependence Index in Population Assessment of Tobacco and Health adult survey (range 1-5; higher scores indicate greater dependence).

^c^ENDS: electronic nicotine delivery systems. Denominator is 481 past-30-day ENDS users with the exception of ever ENDS use.

^d^Participants selected the single flavor, nicotine concentration, or ENDS device they used most often.

### Analysis of Baseline Characteristics by Response to Weekly Surveys

In each flavor group, over 85% of participants who were enrolled and started the 6-week actual use period completed the week 6 survey (Virginia Tobacco, 207/242, 85.5%; Polar Menthol, 212/239, 88.7%; Autumn Tobacco, 190/219, 86.8%; Summer Menthol, 211/236, 89.4%; Ruby Menthol, 201/224, 89.7%) and over 70% completed all 6 weekly surveys (Virginia Tobacco, 178/242, 73.6%; Polar Menthol, 173/239, 72.4%; Autumn Tobacco, 158/219, 72.1%; Summer Menthol, 171/236, 72.5%; Ruby Menthol, 157/224, 70.1%). In the overall sample, there were a few statistically significant differences between the participants who completed all 6 weekly surveys and those who missed some surveys, and the magnitude of differences were small (Multimedia Appendix 7). Participants who completed all 6 surveys (vs 1-5 surveys) were significantly older, by 1.5 years (39.84 vs 38.34 years; *P*=.04), more likely to be female (481/837, 57.5% vs 160/323, 49.5%; *P*=.01), less likely to smoke menthol cigarettes (599/836, 71.7% vs 251/323, 77.7%; *P*=.04) and less likely to have ever used ENDS (575/837, 68.7% vs 250/323, 77.4%; *P*=.003). Among participants who had used ENDS, those who completed all 6 surveys initiated ENDS use about 2 years later (age 31.87 vs 30.01 years; *P*=.03). There were no significant differences by race or ethnicity, education, income, employment, plans to quit smoking, use of JUUL ENDS, or ENDS dependence among those using ENDS. Importantly, there were no differences in the heaviness of smoking or in cigarette dependence, which have been shown to be important predictors of switching. There were no significant differences in rates of survey completion among the JUUL2 flavor groups.

### Switching Across 6-Week Actual Use Period

**Figure 1:**
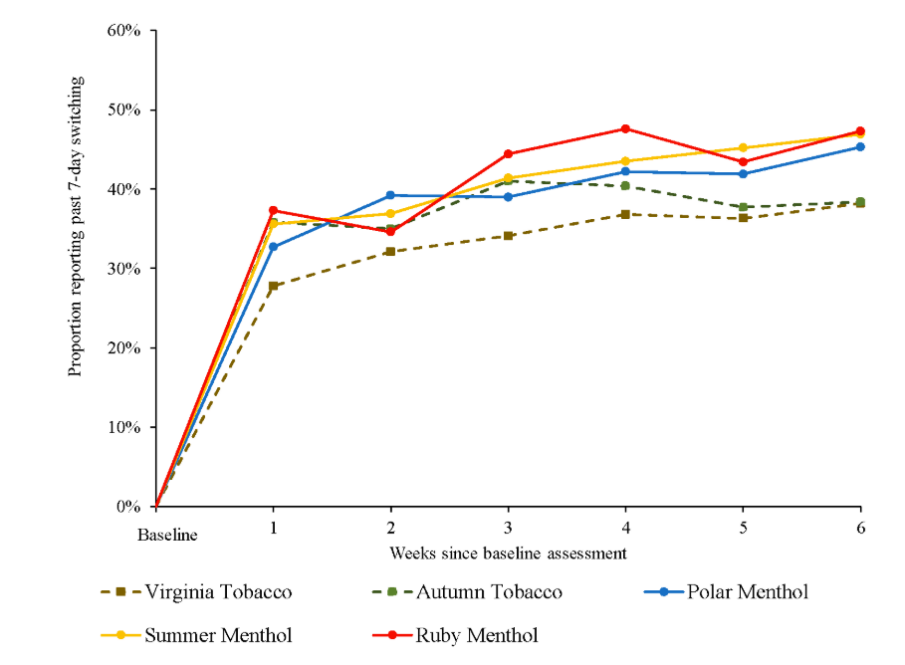
Rates of past 7-day switching away from cigarettes across 6-week actual use period by JUUL2 flavor. Virginia Tobacco: week 1: n=223, week 2: n=234, week 3: n=223, week 4: n=220, week 5: n=215, week 6: n=207; Autumn Tobacco: week 1: n=204, week 2: n=206, week 3: n=200, week 4: n=203, week 5: n=191, week 6: n=190; Polar Menthol: week 1: n=223, week 2: n=222, week 3: n=213, week 4: n=218, week 5: n=210, week 6: n=212; Summer Menthol: week 1: n=216, week 2: n=217, week 3: n=210, week 4: n=214, week 5: n=210, week 6: n=211; Ruby Menthol: week 1: n=204, week 2: n=211, week 3: n=205, week 4: n=206, week 5: n=196, week 6: n=201.

As displayed in Figure 1, in each of the 5 JUUL2 flavor groups, rates of past-7-day switching away from cigarettes increased across the 6-week actual use period: from 27.8% (62/223) at week 1 to 38.2% (79/207) at week 6 for Virginia Tobacco, 35.8% (73/204) at week 1 to 38.4% (73/190) at week 6 for Autumn Tobacco, 32.7% (73/223) at week 1 to 45.3% (96/212) at week 6 for Polar Menthol, 35.6% (77/216) at week 1 to 46.9% (99/211) at week 6 for Summer Menthol, and 37.3% (76/204) at week 1 to 47.3% (95/201) at week 6 for Ruby Menthol. In the combined sample that included all 5 flavors, there was a statistically significant linear increase in the rate of past-7-day switching over the 6-week actual use period (odds ratio [OR] 1.09, 95% CI 1.07-1.12; Multimedia Appendix 8).

At week 6, rates of past-30-day switching were 24.3% (55/226) for Virginia Tobacco, 28.6% (58/203) for Autumn Tobacco, 31.4% (70/223) for Polar Menthol, 33% (69/209) for Ruby Menthol, and 33.9% (74/218) for Summer Menthol (Table 2).

Sensitivity analyses in which smoking (ie, not being switched) was imputed to missing observations showed very similar results, although the absolute imputed switch rates were slightly lower (Multimedia Appendix 9). At week 6, the rates of past-7-day switching using the intent-to-treat approach ranged from 32.6% (79/242; Virginia Tobacco group) to 42.4% (95/224; Ruby Menthol group) and the rates of past-30-day switching ranged from 22.7% (55/242, Virginia Tobacco group) to 31.4% (74/236, Summer Menthol group).

**Table 2:** Rates of past-30-day switching at week 6 by JUUL2 product flavor.

JUUL2 product flavor	Proportion reporting past-30-day switching, % (SE; 95% CI)
Virginia Tobacco (N=226)	24.3 (2.9; 18.7-29.9)
Autumn Tobacco (N=203)	28.6 (3.2; 22.4-34.8)
Polar Menthol (N=223)	31.4 (3.1; 25.3-37.5)
Summer Menthol (N=218)	33.9^a^ (3.2; 27.7-40.2)
Ruby Menthol (N=209)	33^a^ (3.3; 26.6-39.4)

^a^Switch rate is significantly higher than Virginia Tobacco group (*P*<.05).

### Association of JUUL2 Flavor Group and Menthol Cigarette Smoking With Switching

In the model assessing past-30-day switching at week 6, participants who used menthol-flavored (vs tobacco-flavored) JUUL2 products had statistically significant higher switch rates (OR 1.36, 95% CI 1.04-1.78; Multimedia Appendix 8)—there was no significant main effect of cigarette flavor (*P*=.06) and the interaction of JUUL2 flavor group and cigarette flavor was not significant (*P*=.10). Compared to the Virginia Tobacco group, rates of past-30-day switching were significantly higher in the Ruby Menthol group (OR 1.53, 95% CI 1.01-2.33) and Summer Menthol group (OR 1.60, 95% CI 1.06-2.42), respectively. These associations remained significant in the models that imputed smoking for missing data: Ruby Menthol versus Virginia Tobacco (OR 1.51, 95% CI 1.00-2.29) and Summer Menthol versus Virginia Tobacco (OR 1.55, 95% CI 1.03-2.33). The rate of past-30-day switching was numerically higher among the participants in the Polar Menthol group compared to the Virginia Tobacco group, but it did not show a statistically significant difference (OR 1.42, 95% CI 0.94-2.15).

**Figure 2:**
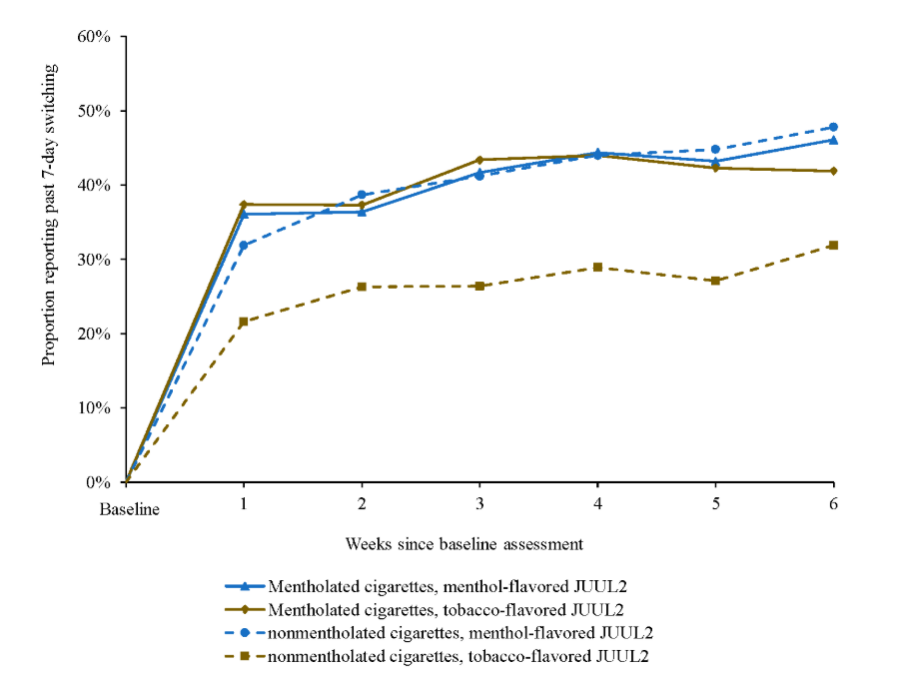
Past 7-day switching by JUUL2 product flavor and cigarette flavor across 6-week actual use period. Smoked menthol cigarettes, menthol-flavored JUUL2: week 1: n=499, week 2: n=508, week 3: n=492, week 4: n=504, week 5: n=482, week 6: n=490; smoked nonmentholated cigarettes, menthol-flavored JUUL2: week 1: n=144, week 2: n=142, week 3: n=136, week 4: n=134, week 5: n=134, week 6: n=134; smoked menthol cigarettes, tobacco-flavored JUUL2: week 1: n=273, week 2: n=287, week 3: n=274, week 4: n=273, week 5: n=265, week 6: N=258; smoked nonmentholated cigarettes, tobacco-flavored JUUL2: week 1: n=153, week 2: n=152, week 3: n=148, week 4: n=149, week 5: n=140, week 6: n=138.

The association of JUUL2 product flavor group and the rate of past-7-day switching was not significant as a main effect (OR 1.23, 95% CI 0.92-1.64; Multimedia Appendix 8). However, this association was significantly moderated by preferred cigarette flavor: among participants who smoked nonmentholated cigarettes, those who used menthol-flavored (vs tobacco-flavored) JUUL2 products had significantly higher switch rates (340/824, 41.3% vs 237/880, 26.9%; OR 1.71, 95% CI 1.04-2.83; Figure 2), whereas there was no significant difference in switch rates by JUUL2 product flavor among participants who smoked menthol cigarettes (1228/2975, 41.3% vs 668/1630, 41%; OR 0.88, 95% CI 0.65-1.18).

There was also a significant main effect of menthol cigarette smoking, with smokers of mentholated (vs nonmentholated) cigarettes demonstrating higher switch rates (OR 1.52, 95% CI 1.14-2.04; Multimedia Appendix 8), largely because of lower switch rates among participants who smoked nonmentholated cigarettes and used tobacco-flavored JUUL2 products (Figure 2). Among participants who smoked mentholated cigarettes, there were no statistically significant differences in sociodemographic or tobacco product use characteristics between those who selected tobacco-flavored and those who selected menthol-flavored JUUL2 products (Multimedia Appendix 10).

### Differences in Switching by ENDS Use at Baseline

**Figure 3:**
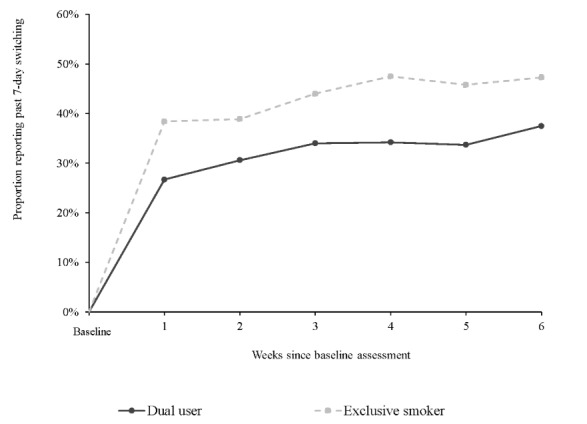
Past 7-days switching among baseline dual users and exclusive smokers across 6-week actual use period. Dual users: week 1: n=430, week 2: n=448, week 3: n=435, week 4: n=433, week 5: n=413, week 6: n=416; exclusive smokers: week 1: n=640, week 2: n=642, week 3: n=616, week 4: n=628, week 5: n=609, week 6: n=605.

There were significant differences in the rates of past-7-day switching according to ENDS use in the 30 days preceding baseline: participants who were smoking but did not use ENDS in the past 30 days had significantly higher switch rates than the dual users (1630/3740, 43.6% vs 843/2575, 32.7%) across the 6-week actual use period (Figure 3 and Multimedia Appendix 11).

### Changes in Dependence and Respiratory Symptoms Among Participants Who Completely Switched (Did Not Smoke) in the Past 30 Days at Week 6

**Figure 4:**
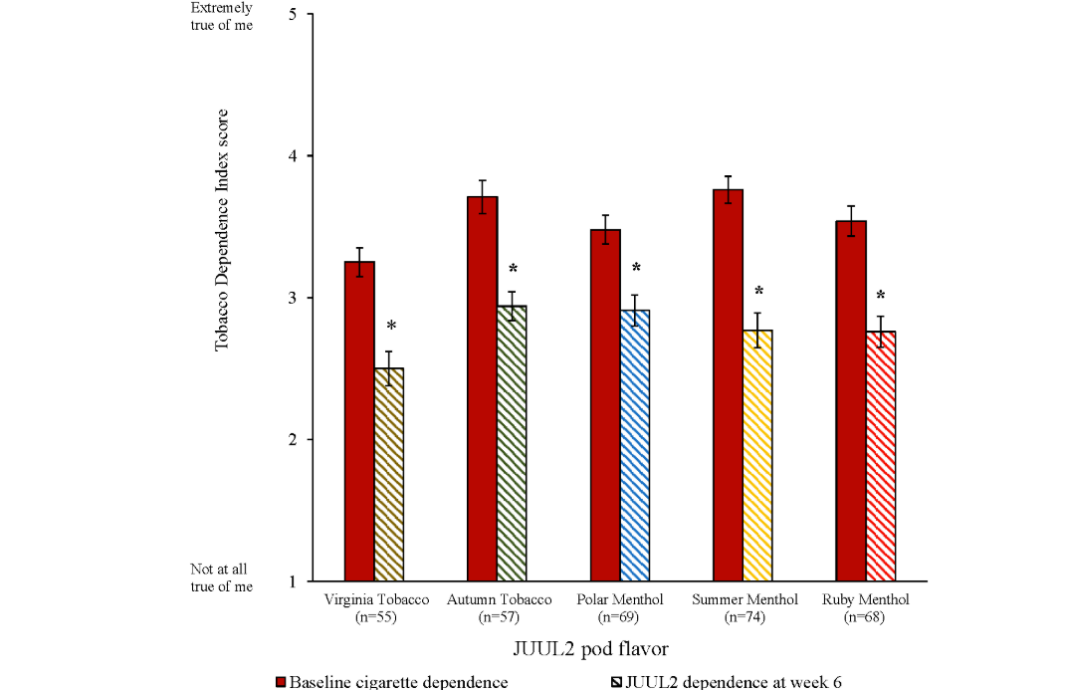
Change from baseline cigarette dependence to JUUL2 dependence at week 6 among the past-30-day switchers (mean, SE). *Statistically significant difference between levels of dependence on cigarettes at baseline and levels of dependence on JUUL2 products at week 6 (*P*<.001).

Among participants who reported past-30-day switching at week 6, levels of dependence on JUUL2 products were significantly lower than their own levels of dependence on combustible cigarettes at baseline, for all 5 flavors (*P*<.001; Figure 4).

**Figure 5:**
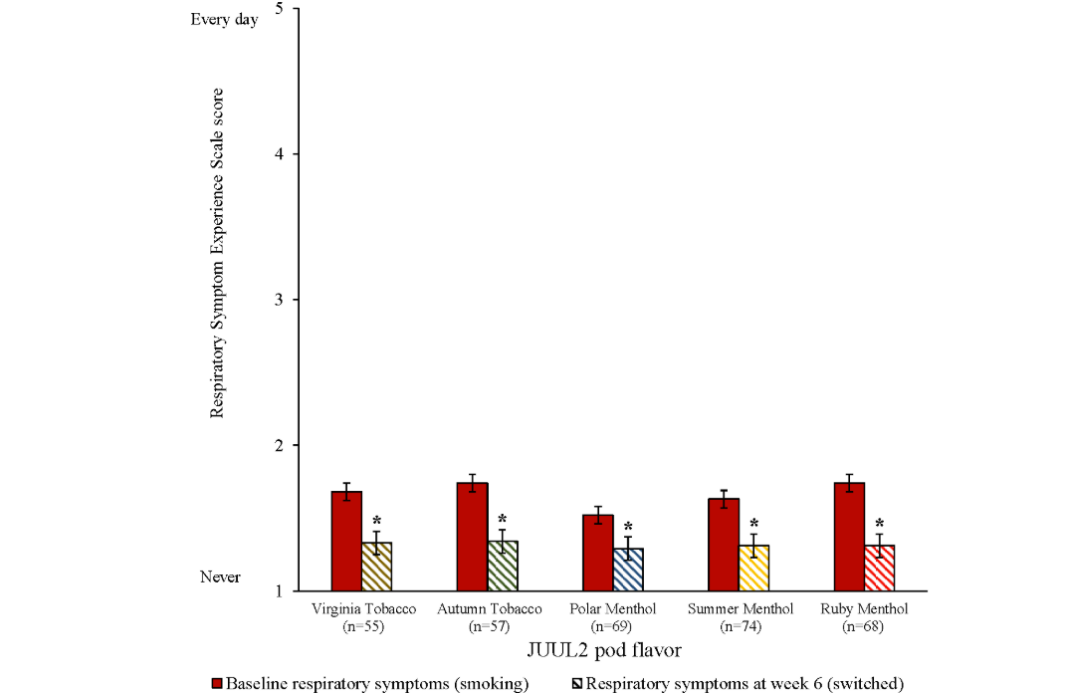
Change in frequency of respiratory symptoms from baseline to week 6 among the past-30-day switchers (mean, SE). *Statistically significant difference between frequency of respiratory symptoms at baseline and at follow-up (P≤.01).

Participants who did not smoke in the past 30 days at week 6 also experienced significant decreases in frequency of self-reported respiratory symptoms relative to baseline when they were smoking cigarettes, across all 5 flavors (*P≤*.01; Figure 5).

### Smoking Reduction Among Participants Who Continued Smoking at Week 6

**Figure 6:**
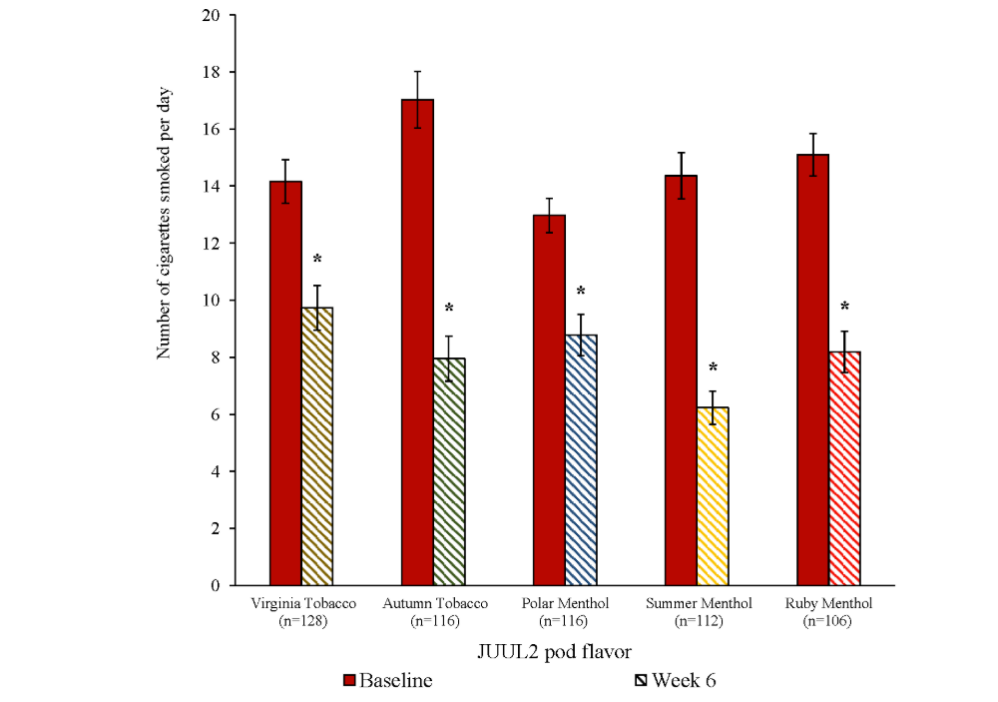
Change in daily cigarette consumption from baseline to week 6 among participants who did not switch (mean, SE). *Statistically significant difference in number of cigarettes per day smoked at baseline and week 6 among participants who continued smoking (*P*<.001).

Among participants who continued to smoke at week 6, average daily cigarette consumption in all 5 JUUL2 flavor groups was significantly reduced relative to participants’ own cigarette consumption at baseline (*P*<.001; Figure 6); across all flavor groups, the proportion of participants that reported reducing their daily cigarette consumption by at least 50% at week 6 relative to baseline ranged from 50.9% (59/116) to 62.9% (73/116; Multimedia Appendix 12).

### Patterns of JUUL2 Product Use and Subjective Effects

**Figure 7:**
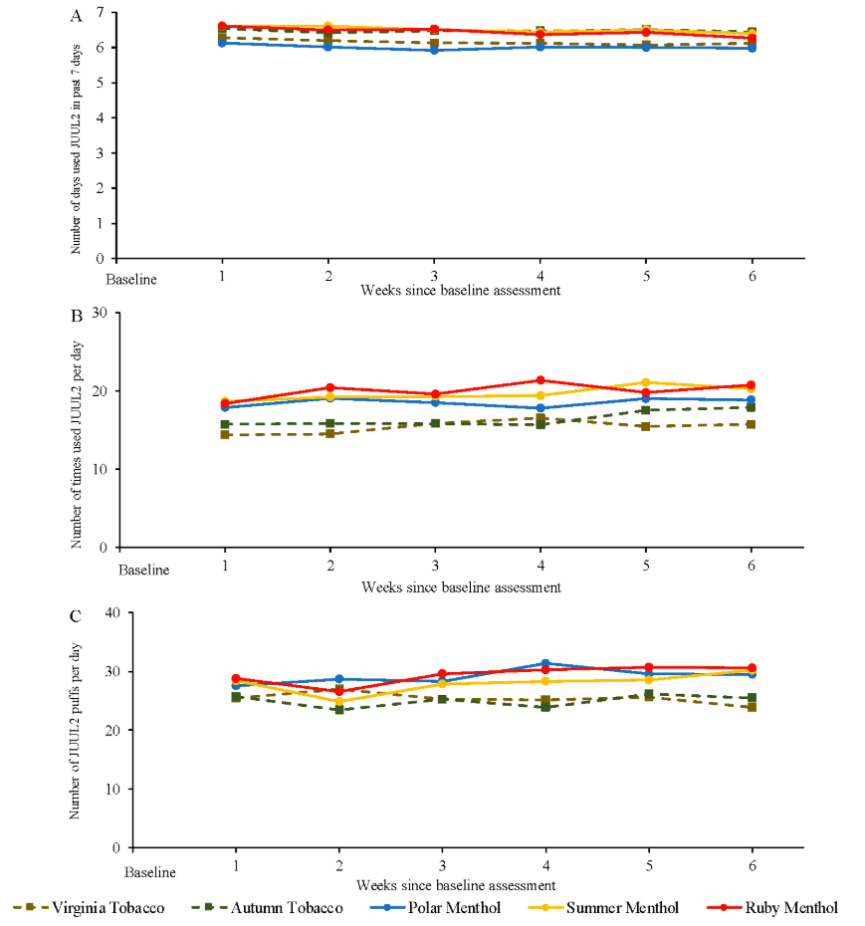
Patterns of JUUL2 product use across 6-week actual use period. (A) Number of days used JUUL2 in past 7 days; (B) number of times used JUUL2 per day; and (C) number of JUUL2 puffs per day.

Across the 6-week actual use period, the prevalence of past-7-day JUUL2 product use was over 97% in each of 5 flavor groups (Multimedia Appendix 13). In each of the 5 flavor groups, the median frequency of JUUL2 product use at each weekly survey was 7 days of use in the past 7 days (ie, daily use; Figure 7A); participants generally reported using JUUL2 products between 15 and 20 times per day (Figure 7B) and 25 to 30 puffs per day (Figure 7C). Across the 6-week period, there were no statistically significant linear increases in JUUL2 use occasions per day in the Virginia Tobacco and Polar Menthol JUUL2 groups (*P*>.15; Multimedia Appendix 14); there were statistically significant linear increases over time in use occasions per day among participants who used Autumn Tobacco, Summer Menthol, and Ruby Menthol JUUL2 products but the magnitude of the increases was small (unstandardized β coefficients=.45−.54; Multimedia Appendix 14). Across the 6-week period, there were no statistically significant increases in JUUL2 puffs per day in the Virginia Tobacco and Autumn Tobacco JUUL2 groups (*P*>.09); there were statistically significant linear increases over time in puffs per day among participants who used Polar Menthol, Summer Menthol, and Ruby Menthol JUUL2 products but the magnitude of the increases was small (unstandardized β coefficients=.43−.60; Multimedia Appendix 14).

Subjective responses to the 5 JUUL2 products were similar across 5 flavors groups at the week 6 survey: ratings of satisfaction approximated “A lot,” ratings of psychological reward approximated “Moderately,” ratings of aversion approximated “Very little,” and ratings of relief approximated “A lot” (Multimedia Appendix 15). When combined across all 5 flavors, ratings of subjective satisfaction (ie, modified Product Evaluation Scale Satisfaction subscale) at week 2 were significantly associated with higher likelihood of complete past-30-day switching at week 6 (OR 1.25, 95% CI 1.11-1.40).

### Adverse Events

The incidence of study-emergent AEs was low for each of the 5 JUUL2 products during the 6-week actual use period—in each group less than 2.5% of participants reported an AE (Virginia Tobacco, 1/262, 0.4%; for, Autumn Tobacco, 2/237, 0.8%, Polar Menthol, 2/262, 0.8%, Ruby Menthol 2/234, 0.9%, for Summer Menthol, 6/249, 2.4%; Multimedia Appendices 16 and 17). No serious AEs were reported in the study and all evaluable AEs were reported as mild or moderate in intensity and resolved during the study.

## Discussion

### Principal Findings

This actual use study enrolled a sample of >1000 US adults who smoked cigarettes daily, following them for 6 weeks as they used JUUL2 ENDS with their preferred flavor in their natural environment. The sample was sociodemographically diverse and predominantly included US adults who smoked mentholated cigarettes, were not ready to quit smoking (ie, were not planning to quit within 30 days), and had experience using ENDS. On average, participants were highly dependent on cigarettes when entering the study. The results demonstrate that the use of JUUL2 ENDS products was associated with high rates of completely switching away from cigarettes among US adults who smoke cigarettes, including both those who smoke mentholated cigarettes and nonmentholated cigarettes and users and nonusers of ENDS. Moreover, participants who reported past-30-day switching at the end of the study experienced meaningful reductions in dependence on tobacco products and improvements in respiratory symptoms during this 6-week period.

Despite a profile that did not favor smoking cessation (eg, high levels of cigarette dependence, almost no participants intending to quit smoking in 30 days, and many never planning to quit), rates of 7-day point prevalence abstinence exceeded 40% after 6 weeks, and at week 6 over 24% of participants in each of the 5 flavor groups (Virginia Tobacco, 55/226, 24.3%; Autumn Tobacco, 58/203, 28.6%, Polar Menthol, 70/223, 31.4%, Ruby Menthol, 69/209, 33%, Summer Menthol, 74/218, 33.9%) reported they had not smoked in the previous 30 days—a criterion often used to evaluate smoking cessation in clinical trials [25]. The proportion reporting switching away from smoking increased over the 6-week period, a pattern that was observed to continue over longer periods in previous observational and randomized studies of JUUL product users [8,26]. Furthermore, the high rates of switching are consistent with participants’ reports of high levels of subjective satisfaction in this study and in experimental studies evaluating JUUL2 products [17], which is an established predictor of switching behavior [26] that was confirmed in this study.

It was notable that high switching rates were observed even though participants reported relatively light use of JUUL2, reportedly averaging around 25 to 30 puffs per day. One factor for this observation may be that the participants appear to have spread their use over time, engaging in “grazing” [27]—they reported averaging 15 to 20 use occasions per day, implying just 1 or 2 puffs per occasion. The volume of aerosol produced by the JUUL2 device may also have made puffs more impactful, mitigating a need for more puffs. In any case, more research on patterns of use associated with switching, including using objective measures of puff topography, would be useful.

Participants who used menthol-flavored (vs tobacco-flavored) JUUL2 products had significantly higher rates of past-30-day switching at the end of the 6-week actual use period. In addition, the association of JUUL2 flavor group and past-7-day switching significantly varied by menthol cigarette smoking (ie, the JUUL2 flavor and menthol cigarette interaction term was statistically significant): participants who smoked nonmentholated cigarettes and used menthol-flavored (vs tobacco-flavored) JUUL2 products demonstrated significantly higher switch rates, but this was not true for those who smoked mentholated cigarettes. The finding that participants who smoked nonmentholated cigarettes and used menthol-flavored ENDS had higher switch rates than those using tobacco-flavored ENDS is consistent with observational studies of US adults who smoke and use ENDS, including JUUL products [28,29], and an actual use study that evaluated the use of heated tobacco products [15]. This suggests that the availability of menthol-flavored ENDS may particularly benefit adults who smoke nonmentholated cigarettes.

Participants who smoked cigarettes but did not use ENDS when they entered the study had significantly higher rates of past-7-day switching compared to those who were dual users (ie, used ENDS and smoked cigarettes concurrently) across the 6-week actual use period. Ongoing dual use may be a behavioral marker for difficulty in stopping smoking with ENDS, as the dual users were, on average, relatively frequent ENDS users at baseline but had not yet completely switched. Nonetheless, both baseline dual users and baseline non-ENDS users displayed high rates of past-7-day switching at the end of the study.

Among participants who reported switching for the past 30 days at week 6, levels of dependence on JUUL2 products were statistically significant and meaningfully lower than participants’ own levels of dependence on cigarettes when they entered the study; the decrease exceeded the minimally important difference for the measure identified in a prior observational study [30]. This finding concords with a large body of evidence indicating that ENDS, including JUUL products, produce lower levels of dependence than cigarettes [1,30-32]. In addition, consumption of JUUL2 products minimally increased across the 6-week actual use period (increases of less than one use occasion and puff per week). In accordance with data from controlled laboratory studies [17], these results demonstrate that JUUL2 products have lower abuse liability than cigarettes.

Participants who completely switched to JUUL2 products for at least 30 days at the completion of the study also reported significantly lower frequency of respiratory symptoms relative to when they entered the study and were smoking cigarettes. This improvement in respiratory symptoms was statistically significant but modest in magnitude—below the minimally important difference of 0.57 proposed in a prior psychometric validation study (operationalized as the difference in frequency of respiratory symptoms between participants with vs without respiratory symptom-relevant diagnoses, including chronic obstructive pulmonary disease) [21]. However, these improvements in respiratory symptoms occurred after relatively short periods of smoking abstinence and 6 weeks of JUUL2 product use, thus it seems likely that respiratory symptoms will continue to improve over longer periods of switching. For example, a study of adults who switched to JUUL products for an average of 3 years found that they had meaningfully lower respiratory symptoms than a matched comparison group that had continued smoking [33]. In this study, the fact that participants reported low baseline respiratory symptom scores when entering the study (mean <2; approximating ratings of “rarely [1-5 days]”), suggested that changes were limited by floor effects.

Substantial proportions of participants who persisted in smoking (ie, were dual users) after 6 weeks of being provided with JUUL2 products significantly reduced their daily cigarette consumption by at least 50% compared to when they entered the study. These levels of reductions in cigarette consumption have been shown to meaningfully reduce exposure to toxicants in cigarette smoke that are associated with smoking-related diseases [34-36] and have been suggested to reduce risk of smoking-related disease [37]. Finally, the JUUL2 products were well tolerated, producing only infrequent and minor AEs.

### Strengths and Limitations

Strengths of the study include the large sociodemographically diverse sample of US adults who smoked every day and reported high levels of cigarette dependence, including large numbers of participants who were not planning to ever quit smoking and smoked mentholated cigarettes. Allowing participants to select their preferred flavor for use during the 6-week actual use period replicated real-world consumer product use and allowed attribution of switching behavior to a single JUUL2 product. However, participants were not allowed to change their selected flavor during the 6-week actual use period, as they would be able to in the real world. In addition, this self-selection of flavors and lack of randomization should be considered when interpreting differences in switch rates between the flavor groups.

Rates of retention were high throughout the 6-week actual use period. In addition, comparisons of sociodemographic and tobacco product characteristics among participants who completed all 6 surveys (vs those who missed some surveys) showed that participants who missed some surveys were not meaningfully different from those who completed all the surveys, and were not heavier or more dependent smokers who would have been expected to have lower rates of switching, suggesting low likelihood of bias due to nonresponse and missing observations. Analyses imputing smoking to all missing observations were performed, and yielded results that paralleled those based on observed data, indicating a lack of material bias in survey response.

Only individuals who reported sufficient levels of interest in using JUUL2 products were eligible to enroll in the study, which may have led to higher switch rates, but also enhanced ecological validity by mirroring real-world use of consumer products, where people buy and adopt products they are interested in, and are not arbitrarily prescribed or assigned products in which they have little interest. Assignment to individual flavors was based on expressed interest, which again is consistent with real-world use. In any case, most participants in both study arms expressed sufficient interest in all the flavors offered to them. Despite this interest in multiple flavors, most of the participants did show substantial interest in one or more flavors offered to them. This is important, as it suggests that making a range of flavors available is likely to increase the proportion of adults who smoke adopt ENDS, thus increasing reach of ENDS and potential switching away from smoking.

Participants were provided with JUUL2 study products at no charge, which is not consistent with the real world, and could have resulted in heavier product use and greater retention or influenced switching. The actual use portion of the study was only 6 weeks in duration and there was no follow-up assessment after completion. Future studies could evaluate trajectories of switching among adults who smoke and adopt JUUL2 products over longer periods. A large study of real-world US adult JUUL purchasers showed that switch rates continued to increase over time, reaching 58% after 2 years of initial purchase [6].

A limitation was the analyses’ reliance on self-report; cigarette smoking and JUUL2 product use were not biochemically verified, and respiratory symptoms were evaluated with a self-report measure. Although this is standard practice for actual use studies [12-14] and observational population-based studies more broadly [24], biomarker data could be useful in future studies. In addition, since the primary aim was descriptive (ie, assessing the proportion of smokers who switched), and did not involve any inferential tests, no adjustment was made for multiplicity. Causal inferences regarding switching are limited by the lack of a control group that did not receive JUUL2 products, hence it is not possible to determine how many participants would have switched or stopped smoking if they were not provided with JUUL2 products. However, almost all participants in the sample did not plan to quit smoking in the next 30 days at baseline, and many were not planning to ever quit, suggesting that quit rates would have been very low.

### Conclusions

The data from this 6-week actual use study demonstrate that adoption of JUUL2 products is associated with substantial rates of completely switching away from cigarettes among US adults who smoke, and meaningful reductions in cigarette consumption among adults who do not completely switch after 6 weeks. Participants who completely switched from smoking cigarettes to use of JUUL2 products at the end of 6 weeks experienced significant reductions in dependence and improvements in respiratory symptoms. Switch rates were higher among participants who used menthol-flavored (vs tobacco-flavored) JUUL2 products, particularly among adults who smoke nonmentholated cigarettes. Although this study primarily focused on behavioral end points, when considered with data from clinical biomarker studies, these changes in tobacco product use behaviors (ie, complete switching and substantial smoking reduction) are likely to decrease exposure to harmful cigarette-related toxicants, and to ultimately reduce risk of tobacco-related diseases.

## Appendix

Multimedia Appendix 1 A schematic diagram of the study design.

Multimedia Appendix 2 The study sites in traditional and complex flavor arms.

Multimedia Appendix 3 Participant flowchart—traditional flavors study arm.

Multimedia Appendix 4 Participant flowchart—complex flavors study arm.

Multimedia Appendix 5 Ratings of interest in JUUL2 products and flavors at the end of trial week.

Multimedia Appendix 6 Sociodemographic and tobacco use characteristics among JUUL2 flavor groups.

Multimedia Appendix 7 Sociodemographic and tobacco use characteristics by number of assessments completed.

Multimedia Appendix 8 Association of JUUL2 flavor group and cigarette flavor with past 7-day and past 30-day switch rates across 6-week actual use period.

Multimedia Appendix 9 Rates of past 30-day and past 7-day switching away from cigarettes across 6-week actual use period by JUUL2 flavor imputing missing as smoking (intent-to-treat).

Multimedia Appendix 10 Sociodemographic and tobacco use characteristics of participants who smoked nonmentholated cigarettes by JUUL2 flavor selection.

Multimedia Appendix 11 Association of past 30-day electronic nicotine delivery systems use at baseline with past 7-day switch rates across 6-week actual use period.

Multimedia Appendix 12 Change in daily cigarette consumption from baseline to week 6 among JUUL2 flavor groups.

Multimedia Appendix 13 Patterns of past 7-day JUUL2 product use across 6-week actual use period among JUUL2 flavor groups.

Multimedia Appendix 14 Temporal trends in daily JUUL2 product use across 6-week actual use period among JUUL2 flavor groups.

Multimedia Appendix 15 Subjective responses to JUUL2 products at week 6 survey among JUUL2 flavor groups.

Multimedia Appendix 16 Adverse events—traditional and complex flavors trial week.

Multimedia Appendix 17 Adverse events over 6-week actual use period among JUUL2 flavor groups.

## Abbreviations

AE: adverse event
ENDS: electronic nicotine delivery systems
OR: odds ratio
