# Supplementary material for: Evaluating the Effect of the JUUL2 System With 5 Flavors on Cigarette Smoking and Tobacco Product Use Behaviors Among Adults Who Smoke Cigarettes: 6-Week Actual Use Study
Source: Interact J Med Res. 2025 Mar 26;14:e60620. doi: 10.2196/60620 (PMC11982753; doi:10.2196/60620)
Supplement: Multimedia Appendix 1 [file ijmr_v14i1e60620_app1.pdf]

Six-Week Actual Use Study to Evaluate the Effect of the JUUL2 System in Five Flavors on Cigarette Smoking and Tobacco Product Use Behaviors among US Adults who Smoke

Multimedia Appendix 1. Schematic of Study Design

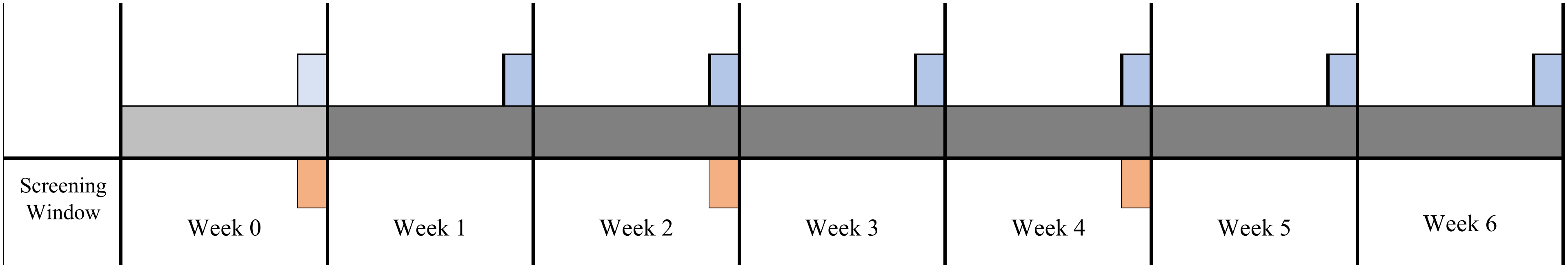

Note. Screening took place no more than 21 days prior to enrollment.

= Product trial period.

= *Ad libitum* product use.

= End of trial assessment and selection of JUUL2 product flavor (evaluation of continued interest in using study product).

= Weekly Assessment.

= Product distribution (20 pods, 10 pods/week).
