# Supplementary material for: Evaluating the Effect of the JUUL2 System With 5 Flavors on Cigarette Smoking and Tobacco Product Use Behaviors Among Adults Who Smoke Cigarettes: 6-Week Actual Use Study
Source: Interact J Med Res. 2025 Mar 26;14:e60620. doi: 10.2196/60620 (PMC11982753; doi:10.2196/60620)
Supplement: Multimedia Appendix 2 [file ijmr_v14i1e60620_app2.pdf]

Six-Week Actual Use Study to Evaluate the Effect of the JUUL2 System in Five Flavors on Cigarette Smoking and Tobacco Product Use Behaviors among US Adults who Smoke

Multimedia Appendix 2. Study Sites in Traditional and Complex Flavor Arms

City	State	Study Flavor Arm
Fort Smith	AR	Complex Flavors
Broomfield	CO	Complex Flavors
Milford	CT	Complex Flavors
Sebring	FL	Complex Flavors
Tallahassee	FL	Complex Flavors
Tampa	FL	Complex Flavors
Alpharetta	GA	Complex Flavors
Schaumburg	IL	Complex Flavors
St. Peters	MO	Complex Flavors
Henderson	NV	Complex Flavors
North Olmsted	OH	Complex Flavors
Humble	TX	Complex Flavors
San Antonio	TX	Complex Flavors
Auburn	WA	Complex Flavors
Chandler	AZ	Traditional Flavors
Pembroke Pines	FL	Traditional Flavors
Ormond Beach	FL	Traditional Flavors
North Riverside	IL	Traditional Flavors
Independence	MO	Traditional Flavors
Charlotte	NC	Traditional Flavors
St. Clairsville	OH	Traditional Flavors
Philadelphia	PA	Traditional Flavors
Grapevine	TX	Traditional Flavors
Fredericksburg	VA	Traditional Flavors

Note. Complex Flavors = Autumn Tobacco, Summer Menthol, Ruby Menthol.
Traditional Flavors = Virginia Tobacco, Polar Menthol.
