# Supplementary material for: Evaluating the Effect of the JUUL2 System With 5 Flavors on Cigarette Smoking and Tobacco Product Use Behaviors Among Adults Who Smoke Cigarettes: 6-Week Actual Use Study
Source: Interact J Med Res. 2025 Mar 26;14:e60620. doi: 10.2196/60620 (PMC11982753; doi:10.2196/60620)
Supplement: Multimedia Appendix 5 [file ijmr_v14i1e60620_app5.pdf]

Multimedia Appendix 5. Ratings of Interest in JUUL2 Products and Flavors at End of Trial Week

JUUL2 pod Flavor Group	Purchase Interest[a] Mean (*SD*)	Flavor Interest[b] Mean (*SD*)	All Flavors Ranked Equally N (%)	Equal Preference for Two Flavors N (%)	Preference for One Flavor N (%)
Virginia Tobacco (N=574)	3.37 (0.68)	3.65 (1.25)	—	—	—
Polar Menthol (N=576)	3.37 (0.68)	4.12 (0.98)	—	—	—
Autumn Tobacco (N=797)	3.44 (0.68)	3.60 (1.27)	—	—	—
Summer Menthol (N=798)	3.43 (0.68)	3.91 (1.11)	—	—	—
Ruby Menthol (N=800)	3.44 (0.68)	3.94 (1.12)	—	—	—
JUUL2 Study Arm					
Traditional Flavors Arm (N=574)	—	—	242 (42.2%)	—	332 (57.8%)
Complex Flavors Arm (N=792)	—	—	232 (29.3%)	216 (27.3%)	344 (43.4%)
