# Supplementary material for: Evaluating the Effect of the JUUL2 System With 5 Flavors on Cigarette Smoking and Tobacco Product Use Behaviors Among Adults Who Smoke Cigarettes: 6-Week Actual Use Study
Source: Interact J Med Res. 2025 Mar 26;14:e60620. doi: 10.2196/60620 (PMC11982753; doi:10.2196/60620)
Supplement: Multimedia Appendix 6 [file ijmr_v14i1e60620_app6.pdf]

Multimedia Appendix 6. Sociodemographic and Tobacco Use Characteristics among JUUL2 Flavor Groups

Sample Characteristics	Virginia Tobacco	Autumn Tobacco	Polar Menthol	Summer Menthol	Ruby Menthol
**Sociodemographic Characteristics**					
Age, yr, *Mean* (*SD*)	39.29 (10.81)	41.21 (11.12)	38.18 (11.41)	38.99 (10.48)	39.59 (11.21)
Sex					
Male	99 (40.9%)	110 (50.2%)	105 (43.9%)	112 (47.5%)	91 (40.6%)
Female	143 (59.1%)	109 (49.8%)	134 (56.1%)	124 (52.5%)	131 (58.5%)
Other	0 (0.0%)	0 (0.0%)	0 (0.0%)	0 (0.0%)	1 (0.4%)
Prefer not to answer	0 (0.0%)	0 (0.0%)	0 (0.0%)	0 (0.0%)	1 (0.4%)
Race/Ethnicity					
Non-Hispanic White	134 (55.4%)	137 (62.6%)	131 (54.8%)	129 (54.7%)	136 (60.7%)
Non-Hispanic Black	55 (22.7%)	31 (14.2%)	61 (25.5%)	42 (17.8%)	34 (15.2%)
Non-Hispanic Other Race	11 (4.5%)	14 (6.4%)	12 (5.0%)	16 (6.8%)	13 (5.8%)
Hispanic Ethnicity	39 (16.1%)	35 (16.0%)	32 (13.4%)	46 (19.5%)	39 (17.4%)
Unknown	3 (1.2%)	2 (0.9%)	3 (1.3%)	3 (1.3%)	2 (0.9%)
Marital Status					
Married	66 (27.3%)	87 (39.7%)	58 (24.3%)	80 (33.9%)	78 (34.8%)
Living with Partner	49 (20.2%)	38 (17.4%)	46 (19.2%)	48 (20.3%)	41 (18.3%)
Divorced, Separated or Widowed	36 (14.9%)	31 (14.2%)	34 (14.2%)	27 (11.4%)	39 (17.4%)
Never Married	88 (36.4%)	60 (27.4%)	96 (40.2%)	75 (31.8%)	60 (26.8%)
Prefer not to say	3 (1.2%)	3 (1.4%)	5 (2.1%)	6 (2.5%)	6 (2.7%)
Annual Household Income					
<$50,000	143 (59.1%)	105 (47.9%)	151 (63.2%)	124 (52.5%)	109 (48.7%)
$50,000-$99,999	74 (30.6%)	96 (43.8%)	70 (29.3%)	84 (35.6%)	84 (37.5%)
$100,000 or more	25 (10.3%)	18 (8.2%)	18 (7.5%)	28 (11.9%)	31 (13.8%)
Highest Level of Education					
High school graduate or less	97 (40.1%)	83 (37.9%)	102 (42.7%)	92 (39.0%)	85 (37.9%)
Some college or trade school	88 (36.4%)	77 (35.2%)	88 (36.8%)	89 (37.7%)	80 (35.7%)
College graduate or more education	57 (23.6%)	59 (26.9%)	49 (20.5%)	55 (23.3%)	59 (26.3%)
Employment Status					
Full time	141 (58.3%)	149 (68.0%)	162 (67.8%)	157 (66.5%)	145 (64.7%)
Part time	29 (12.0%)	31 (14.2%)	26 (10.9%)	30 (12.7%)	29 (12.9%)
Other	72 (29.8%)	39 (17.8%)	51 (21.3%)	49 (20.8%)	50 (22.3%)
Census Region					
Northeast	24 (9.9%)	1 (0.5%)	23 (9.6%)	2 (0.8%)	4 (1.8%)
Midwest	88 (36.4%)	59 (26.9%)	89 (37.2%)	63 (26.7%)	57 (25.4%)
South	114 (47.1%)	109 (49.8%)	118 (49.4%)	146 (61.9%)	119 (53.1%)

West	16 (6.6%)	50 (22.8%)	9 (3.8%)	25 (10.6%)	44 (19.6%)
**Smoking Characteristics**					
Smoke Mentholated Cigarettes	172 (71.1%)	130 (59.6%)	193 (80.8%)	188 (79.7%)	167 (74.6%)
No. Cigarettes Smoked per Smoking Day, *Mean* (*SD*)	13.10 (8.17)	15.57 (12.72)	12.99 (6.80)	14.26 (8.68)	14.81 (7.33)
Duration of Smoking, yr *Mean* (*SD*)	15.71 (10.17)	18.33 (10.13)	14.46 (9.86)	15.93 (9.13)	16.81 (9.70)
Age started smoking, yr, *Mean* (*SD*)	19.64 (6.28)	18.94 (6.61)	19.62 (6.66)	19.31 (5.86)	19.12 (5.91)
Cigarette Dependence[a] *Mean* (*SD*)	3.56 (0.82)	3.70 (0.83)	3.62 (0.79)	3.71 (0.82)	3.65 (0.77)
Plan to Quit Smoking in Next 30 Days	2 (0.8%)	2 (0.9%)	3 (1.3%)	1 (0.4%)	3 (1.3%)
Ever Plan to Quit Smoking	54 (22.3%)	60 (27.4%)	79 (33.1%)	69 (29.2%)	66 (29.5%)
**ENDS Use Characteristics**					
Ever used ENDS	183 (75.6%)	155 (70.8%)	183 (76.6%)	145 (61.4%)	159 (71.0%)
Age First Used ENDS, yr, *Mean* (*SD*)	31.66 (11.13)	33.17 (11.85)	30.64 (12.13)	30.18 (10.40)	30.88 (11.15)
Ever used ENDS Fairly Regularly	123 (67.2%)	93 (60.0%)	122 (66.7%)	91 (62.8%)	100 (62.9%)
Used ENDS in Past 30 Days	123 (67.2%)	72 (46.5%)	129 (70.5%)	76 (52.4%)	81 (50.9%)
No. Days Used ENDS in P30D, *Mean* (*SD*)	15.50 (9.80)	14.67 (10.23)	15.19 (10.16)	16.24 (10.02)	14.59 (8.83)
No. Times Used ENDS per Use Day, *Median* (*IQR*)	8 (10)	10 (11.5)	9 (10)	10 (17)	10 (15)
ENDS Dependence,[a] *Mean* (*SD*)	3.03 (0.94)	3.07 (0.96)	2.99 (0.99)	3.13 (1.08)	2.93 (0.92)
Primary ENDS Flavor[b]					
Tobacco	17 (13.8%)	12 (16.7%)	10 (7.8%)	5 (6.6%)	6 (7.4%)
Menthol	45 (36.6%)	18 (25.0%)	49 (38.0%)	32 (42.1%)	39 (48.1%)
Mint	10 (8.1%)	12 (16.7%)	14 (10.9%)	9 (11.8%)	10 (12.3%)
Fruit	40 (32.5%)	24 (33.3%)	47 (36.4%)	21 (27.6%)	20 (24.7%)
Dessert/Candy	10 (8.1%)	5 (6.9%)	6 (4.7%)	8 (10.5%)	4 (4.9%)
Spice/Clove	0 (0.0%)	0 (0.0%)	1 (0.8%)	0 (0.0%)	1 (1.2%)
Some other flavor	1 (0.8%)	1 (1.4%)	2 (1.6%)	1 (1.3%)	1 (1.2%)
Primary ENDS Device Type[b]					
Pod-based	38 (30.9%)	36 (50.0%)	38 (29.5%)	27 (35.5%)	29 (35.8%)
Disposable	58 (47.2%)	26 (36.1%)	69 (53.5%)	36 (47.4%)	40 (49.4%)
Tank	19 (15.4%)	7 (9.7%)	16 (12.4%)	8 (10.5%)	6 (7.4%)
Mod	3 (2.4%)	0 (0.0%)	2 (1.6%)	2 (2.6%)	1 (1.2%)
Primary ENDS Brand[b]					
JUUL	49 (39.8%)	27 (37.5%)	34 (26.4%)	31 (40.8%)	25 (30.9%)
Vuse	12 (9.8%)	15 (20.8%)	14 (10.9%)	7 (9.2%)	15 (18.5%)
Blu	20 (16.3%)	6 (8.3%)	15 (11.6%)	5 (6.6%)	5 (6.2%)
NJOY	8 (6.5%)	4 (5.6%)	8 (6.2%)	6 (7.9%)	3 (3.7%)
Puff Bar	8 (6.5%)	8 (11.1%)	22 (17.1%)	13 (17.1%)	16 (19.8%)
Other	26 (21.1%)	12 (16.7%)	36 (27.9%)	14 (18.4%)	17 (21.0%)

Note. Values represent N (%) or Mean (SD) unless otherwise noted. Denominators may be less than totals in column heads.

[a] Tobacco Dependence Index in PATH adult survey (Range: 1-5; higher scores indicate greater dependence).
[b] Participants selected the single flavor, nicotine concentration or ENDS device they used most often.
[c] Includes smokeless tobacco and tobacco-free “modern” oral nicotine pouches.
