# Supplementary material for: Evaluating the Effect of the JUUL2 System With 5 Flavors on Cigarette Smoking and Tobacco Product Use Behaviors Among Adults Who Smoke Cigarettes: 6-Week Actual Use Study
Source: Interact J Med Res. 2025 Mar 26;14:e60620. doi: 10.2196/60620 (PMC11982753; doi:10.2196/60620)
Supplement: Multimedia Appendix 7 [file ijmr_v14i1e60620_app7.pdf]

Multimedia Appendix 7. Sociodemographic and Tobacco Use Characteristics by Number of Assessments Completed

Sample Characteristics	N (%) or Mean (*SD*)		*p*-value
	>1 Missing Weekly Surveys (N=323)	No Missed Weekly Surveys (N=837)	
**Flavor Assignment**			
Virginia Tobacco	64 (19.8%)	178 (21.3%)	0.465
Polar Menthol	66 (20.4%)	173 (20.7%)	
Autumn Tobacco	61 (18.9%)	158 (18.9%)	
Ruby Menthol	67 (20.7%)	157 (18.8%)	
Summer Menthol	65 (20.1%)	171 (20.4%)	
**Sociodemographic Characteristics**			
Age, yr, *Mean* (*SD*)	38.34 (11.06)	39.84 (11.00)	0.038
Sex			
Male	162 (50.2%)	355 (42.4%)	0.014
Female	160 (49.5%)	481 (57.5%)	
Other	1 (0.3%)	0 (0.0%)	
Prefer not to answer	0 (0.0%)	1 (0.1%)	
Race/Ethnicity			
Non-Hispanic White	177 (54.8%)	490 (58.5%)	0.246
Non-Hispanic Black	60 (18.6%)	163 (19.5%)	
Non-Hispanic Other Race	24 (7.4%)	42 (5.0%)	
Hispanic Ethnicity	56 (17.3%)	135 (16.1%)	
Unknown	6 (1.9%)	7 (0.8%)	
Marital Status			
Married	84 (26.0%)	285 (34.1%)	0.077
Living with Partner	73 (22.6%)	149 (17.8%)	
Divorced, Separated or Widowed	49 (15.2%)	118 (14.1%)	
Never Married	109 (33.7%)	270 (32.3%)	
Prefer not to say	8 (2.5%)	15 (1.8%)	
Annual Household Income			
<$50,000	168 (52.0%)	494 (55.4%)	0.313
$50,000-$99,999	115 (35.6%)	293 (35.0%)	
$100,000 or more	40 (12.4%)	80 (9.6%)	
Highest Level of Education			
High school graduate or less	130 (40.2%)	329 (39.3%)	0.169
Some college or trade school	127 (39.3%)	295 (35.2%)	
College graduate or more education	66 (20.4%)	213 (25.4%)	
Employment Status			
Full time	203 (62.8%)	551 (65.8%)	0.622
Part time	48 (14.9%)	97 (11.6%)	
Other	72 (22.3%)	189 (22.6%)	
Census Region			
Northeast	8 (2.5%)	46 (5.5%)	0.116
Midwest	94 (29.1%)	262 (31.3%)	
South	179 (55.4%)	427 (51.0%)	
West	42 (13.0%)	102 (12.2%)	
**Smoking Characteristics**			
Smoke Mentholated Cigarettes	251 (77.7%)	599 (71.7%)	0.037
No. Cigarettes Smoked per Smoking Day, *Mean* (*SD*)	14.22 (8.28)	14.07 (9.22)	0.785
Duration of Smoking, yr *Mean* (*SD*)	15.41 (10.04)	16.51 (9.79)	0.088

Age started smoking, yr, *Mean* (*SD*)	19.31 (5.98)	19.34 (6.38)	0.927
Cigarette Dependence[a] *Mean* (*SD*)	3.61 (0.83)	3.66 (0.80)	0.393
Plan to Quit Smoking in Next 30 Days	5 (1.5%)	6 (0.7%)	0.192
Ever Plan to Quit Smoking	92 (28.5%)	236 (28.2%)	0.923
**ENDS Use Characteristics**			
Ever used ENDS	250 (77.4%)	575 (68.7%)	0.003
Age First Used ENDS, yr, *Mean* (*SD*)	30.01 (11.14)	31.87 (11.47)	0.031
Ever used ENDS Fairly Regularly	169 (67.6%)	360 (62.6%)	0.170
Used ENDS in Past 30 Days	139 (55.6%)	342 (59.5%)	0.299
No. Days Used ENDS in P30D, *Mean* (*SD*)	14.80 (9.68)	15.44 (9.88)	0.518
No. Times Used ENDS per Use Day, *Median* (*IQR*)	8 (11)	9.5 (15)	0.532
ENDS Dependence,[a] *Mean* (*SD*)	2.99 (0.99)	3.04 (0.97)	0.619
Primary ENDS Flavor[b]			
Tobacco	14 (10.1%)	36 (10.5%)	0.753
Menthol	47 (33.8%)	136 (39.8%)	
Mint	18 (12.9%)	37 (10.8%)	
Fruit	48 (34.5%)	104 (30.4%)	
Dessert/Candy	11 (7.9%)	22 (6.4%)	
Spice/Clove	0 (0.0%)	2 (0.6%)	
Some other flavor	1 (0.7%)	5 (1.5%)	
Primary ENDS Device Type[b]			
Pod-based	44 (31.7%)	124 (36.3%)	0.738
Disposable	68 (48.9%)	161 (47.1%)	
Tank	17 (12.2%)	39 (11.4%)	
Mod	2 (1.4%)	6 (1.8%)	
Primary ENDS Brand[b]			
JUUL	50 (36.0%)	116 (33.9%)	0.831
Vuse	18 (12.9%)	45 (13.2%)	
Blu	18 (12.9%)	33 (9.6%)	
NJOY	9 (6.5%)	20 (5.8%)	
Puff Bar	17 (12.2%)	50 (14.6%)	
Other	27 (19.4%)	78 (22.8%)	

Note. Values represent N (%) or Mean (SD) unless otherwise noted. Denominators may be less than totals in column heads.

[a] Tobacco Dependence Index in PATH adult survey (Range: 1-5; higher scores indicate greater dependence).

[b] Participants selected the single flavor, nicotine concentration or ENDS device they used most often.

[c] Includes smokeless tobacco and tobacco-free "modern" oral nicotine pouches.

Six-Week Actual Use Study to Evaluate the Effect of the JUUL2 System in Five Flavors on Cigarette Smoking and Tobacco Product Use Behaviors among US Adults who Smoke

Table S12. Adverse Events – Traditional and Complex Flavors Trial Week

Adverse Events (AEs)	Traditional Flavors n (%) [E]*	Complex Flavors n (%) [E]*
Total Participants Reporting at Least One AE	1 (0.2%) [2]	4 (0.4%) [7]
Serious AEs	0 (0.0%) [0]	0 (0.0%) [0]
Death	0 (0.0%) [0]	0 (0.0%) [0]
Life Threatening	0 (0.0%) [0]	0 (0.0%) [0]
Hospitalization	0 (0.0%) [0]	0 (0.0%) [0]
Disabling	0 (0.0%) [0]	0 (0.0%) [0]
Birth Defect	0 (0.0%) [0]	0 (0.0%) [0]
Medically Important	0 (0.0%) [0]	0 (0.0%) [0]
Maximum Intensity		
Severe	0 (0.0%) [0]	0 (0.0%) [0]
Moderate	1 (0.2%) [2]	0 (0.0%) [0]
Mild	0 (0.0%) [0]	4 (0.4%) [7]
Unknown	0 (0.0%) [0]	0 (0.0%) [0]
Worst Relationship		
Related	0 (0.0%) [0]	0 (0.0%) [0]
Probably Related	1 (0.2%) [2]	2 (0.2%) [5]
Possibly Related	0 (0.0%) [0]	1 (0.1%) [1]
Unlikely Related	0 (0.0%) [0]	0 (0.0%) [0]
Not Related	0 (0.0%) [0]	1 (0.1%) [1]
Outcome of AE		
Fatal	0 (0.0%) [0]	0 (0.0%) [0]
Worse	0 (0.0%) [0]	0 (0.0%) [0]
Unchanged	0 (0.0%) [0]	0 (0.0%) [0]
Improved	0 (0.0%) [0]	0 (0.0%) [0]
Resolved	1 (0.2%) [2]	4 (0.4%) [7]
Unknown	0 (0.0%) [0]	0 (0.0%) [0]
MedDRA System Organ Class Preferred Term		
Nervous system disorders	0 (0.0%) [0]	0 (0.0%) [0]
Dizziness	0 (0.0%) [0]	0 (0.0%) [0]
Headache	0 (0.0%) [0]	0 (0.0%) [0]
Somnolence	0 (0.0%) [0]	0 (0.0%) [0]
Syncope	0 (0.0%) [0]	0 (0.0%) [0]
Hypoaesthesia	0 (0.0%) [0]	0 (0.0%) [0]
Gastrointestinal disorders	0 (0.0%) [0]	0 (0.0%) [0]
Abdominal pain upper	0 (0.0%) [0]	0 (0.0%) [0]
Constipation	0 (0.0%) [0]	0 (0.0%) [0]
Diarrhea	0 (0.0%) [0]	0 (0.0%) [0]
Nausea	0 (0.0%) [0]	0 (0.0%) [0]
Vomiting	0 (0.0%) [0]	0 (0.0%) [0]
Respiratory, thoracic, and mediastinal disorders	1 (0.2%) [1]	1 (0.1%) [4]
Cough	0 (0.0%) [0]	1 (0.1%) [1]
Throat irritation	0 (0.0%) [0]	1 (0.1%) [1]
Dysphonia	0 (0.0%) [0]	1 (0.1%) [1]
Dyspnoea	1 (0.2%) [1]	0 (0.0%) [0]
Haemoptysis	0 (0.0%) [0]	1 (0.1%) [1]

Nasal congestion	0 (0.0%) [0]	0 (0.0%) [0]
Productive cough	0 (0.0%) [0]	0 (0.0%) [0]
Skin and subcutaneous tissue disorders	0 (0.0%) [0]	1 (0.1%) [1]
Cold sweat	0 (0.0%) [0]	0 (0.0%) [0]
Hyperhidrosis	0 (0.0%) [0]	0 (0.0%) [0]
Blister	0 (0.0%) [0]	0 (0.0%) [0]
Dermatitis	0 (0.0%) [0]	1 (0.1%) [1]
Dermatitis atopic	0 (0.0%) [0]	0 (0.0%) [0]
Vascular disorders	0 (0.0%) [0]	0 (0.0%) [0]
Hot flush	0 (0.0%) [0]	0 (0.0%) [0]
Pallor	0 (0.0%) [0]	0 (0.0%) [0]
Psychiatric disorders	0 (0.0%) [0]	0 (0.0%) [0]
Anxiety	0 (0.0%) [0]	0 (0.0%) [0]
General disorders and administration site conditions	1 (0.2%) [1]	1 (0.1%) [1]
Chest pain	1 (0.2%) [1]	1 (0.1%) [1]
Unevaluable event	0 (0.0%) [0]	0 (0.0%) [0]
Infections and infestations	0 (0.0%) [0]	0 (0.0%) [0]
Conjunctivitis	0 (0.0%) [0]	0 (0.0%) [0]
Influenza	0 (0.0%) [0]	0 (0.0%) [0]
Upper respiratory tract infection	0 (0.0%) [0]	0 (0.0%) [0]
Respiratory tract infection	0 (0.0%) [0]	0 (0.0%) [0]
COVID-19	0 (0.0%) [0]	0 (0.0%) [0]
Injury, poisoning and procedural complications	0 (0.0%) [0]	1 (0.1%) [1]
Road traffic accident	0 (0.0%) [0]	1 (0.1%) [1]

Traditional Flavors, N=648; Complex Flavors, N=910.

Note. Participants reporting more than one event in a category are counted only once for that category.

*n (%) is the number and percent of participants with AEs and [E] is the total number of AEs reported, as multiple AEs could occur per session.

Table S13. Adverse Events over Six-Week Actual Use Period among JUUL2 Flavor Groups

Adverse Events (AEs)	Virginia Tobacco n (%) [E]*	Autumn Tobacco n (%) [E]*	Polar Menthol n (%) [E]*	Summer Menthol n (%) [E]*	Ruby Menthol n (%) [E]*
Total Participants Reporting at Least One AE	1 (0.4%) [1]	2 (0.8%) [2]	2 (0.8%) [4]	6 (2.4%) [6]	2 (0.9%) [2]
Serious AEs	0 (0.0%) [0]	0 (0.0%) [0]	0 (0.0%) [0]	0 (0.0%) [0]	0 (0.0%) [0]
Death	0 (0.0%) [0]	0 (0.0%) [0]	0 (0.0%) [0]	0 (0.0%) [0]	0 (0.0%) [0]
Life Threatening	0 (0.0%) [0]	0 (0.0%) [0]	0 (0.0%) [0]	0 (0.0%) [0]	0 (0.0%) [0]
Hospitalization	0 (0.0%) [0]	0 (0.0%) [0]	0 (0.0%) [0]	0 (0.0%) [0]	0 (0.0%) [0]
Disabling	0 (0.0%) [0]	0 (0.0%) [0]	0 (0.0%) [0]	0 (0.0%) [0]	0 (0.0%) [0]
Birth Defect	0 (0.0%) [0]	0 (0.0%) [0]	0 (0.0%) [0]	0 (0.0%) [0]	0 (0.0%) [0]
Medically Important	0 (0.0%) [0]	0 (0.0%) [0]	0 (0.0%) [0]	0 (0.0%) [0]	0 (0.0%) [0]
Maximum Intensity					
Severe	0 (0.0%) [0]	0 (0.0%) [0]	0 (0.0%) [0]	0 (0.0%) [0]	0 (0.0%) [0]
Moderate	0 (0.0%) [0]	1 (0.4%) [1]	1 (0.4%) [1]	1 (0.4%) [1]	1 (0.4%) [1]
Mild	0 (0.0%) [0]	1 (0.4%) [1]	2 (0.8%) [3]	5 (2.0%) [5]	1 (0.4%) [1]
Unknown	1 (0.4%) [1]	0 (0.0%) [0]	0 (0.0%) [0]	0 (0.0%) [0]	0 (0.0%) [0]
Worst Relationship					
Related	0 (0.0%) [0]	0 (0.0%) [0]	0 (0.0%) [0]	0 (0.0%) [0]	0 (0.0%) [0]
Probably Related	0 (0.0%) [0]	0 (0.0%) [0]	0 (0.0%) [0]	1 (0.4%) [1]	0 (0.0%) [0]
Possibly Related	0 (0.0%) [0]	0 (0.0%) [0]	1 (0.4%) [2]	0 (0.0%) [0]	1 (0.4%) [1]
Unlikely Related	0 (0.0%) [0]	1 (0.4%) [1]	0 (0.0%) [0]	3 (1.2%) [3]	0 (0.0%) [0]
Not Related	1 (0.4%) [1]	1 (0.4%) [1]	1 (0.4%) [2]	2 (0.8%) [2]	1 (0.4%) [1]
Outcome of AE					
Fatal	0 (0.0%) [0]	0 (0.0%) [0]	0 (0.0%) [0]	0 (0.0%) [0]	0 (0.0%) [0]
Worse	0 (0.0%) [0]	0 (0.0%) [0]	0 (0.0%) [0]	0 (0.0%) [0]	0 (0.0%) [0]
Unchanged	0 (0.0%) [0]	0 (0.0%) [0]	0 (0.0%) [0]	0 (0.0%) [0]	0 (0.0%) [0]
Improved	0 (0.0%) [0]	0 (0.0%) [0]	0 (0.0%) [0]	0 (0.0%) [0]	0 (0.0%) [0]
Resolved	0 (0.0%) [0]	2 (0.8%) [2]	2 (0.8%) [4]	5 (2.0%) [5]	2 (0.9%) [2]
Unknown	1 (0.4%) [1]	0 (0.0%) [0]	0 (0.0%) [0]	1 (0.4%) [1]	0 (0.0%) [0]
MedDRA System Organ Class<br>Preferred Term					
Nervous system disorders	0 (0.0%) [0]	0 (0.0%) [0]	0 (0.0%) [0]	1 (0.4%) [1]	0 (0.0%) [0]
Dizziness	0 (0.0%) [0]	0 (0.0%) [0]	0 (0.0%) [0]	0 (0.0%) [0]	0 (0.0%) [0]
Headache	0 (0.0%) [0]	0 (0.0%) [0]	0 (0.0%) [0]	0 (0.0%) [0]	0 (0.0%) [0]
Somnolence	0 (0.0%) [0]	0 (0.0%) [0]	0 (0.0%) [0]	0 (0.0%) [0]	0 (0.0%) [0]

Syncope	0 (0.0%) [0]	0 (0.0%) [0]	0 (0.0%) [0]	0 (0.0%) [0]	0 (0.0%) [0]
Hypoaesthesia	0 (0.0%) [0]	0 (0.0%) [0]	0 (0.0%) [0]	1 (0.4%) [1]	0 (0.0%) [0]
Gastrointestinal disorders	0 (0.0%) [0]	0 (0.0%) [0]	0 (0.0%) [0]	0 (0.0%) [0]	0 (0.0%) [0]
Abdominal pain upper	0 (0.0%) [0]	0 (0.0%) [0]	0 (0.0%) [0]	0 (0.0%) [0]	0 (0.0%) [0]
Constipation	0 (0.0%) [0]	0 (0.0%) [0]	0 (0.0%) [0]	0 (0.0%) [0]	0 (0.0%) [0]
Diarrhea	0 (0.0%) [0]	0 (0.0%) [0]	0 (0.0%) [0]	0 (0.0%) [0]	0 (0.0%) [0]
Nausea	0 (0.0%) [0]	0 (0.0%) [0]	0 (0.0%) [0]	0 (0.0%) [0]	0 (0.0%) [0]
Vomiting	0 (0.0%) [0]	0 (0.0%) [0]	0 (0.0%) [0]	0 (0.0%) [0]	0 (0.0%) [0]
Respiratory, thoracic, and mediastinal disorders	0 (0.0%) [0]	0 (0.0%) [0]	1 (0.4%) [2]	2 (0.8%) [2]	1 (0.4%) [1]
Cough	0 (0.0%) [0]	0 (0.0%) [0]	0 (0.0%) [0]	0 (0.0%) [0]	1 (0.4%) [1]
Throat irritation	0 (0.0%) [0]	0 (0.0%) [0]	0 (0.0%) [0]	1 (0.4%) [1]	0 (0.0%) [0]
Dysphonia	0 (0.0%) [0]	0 (0.0%) [0]	1 (0.4%) [1]	0 (0.0%) [0]	0 (0.0%) [0]
Dyspnoea	0 (0.0%) [0]	0 (0.0%) [0]	0 (0.0%) [0]	0 (0.0%) [0]	0 (0.0%) [0]
Haemoptysis	0 (0.0%) [0]	0 (0.0%) [0]	0 (0.0%) [0]	0 (0.0%) [0]	0 (0.0%) [0]
Nasal congestion	0 (0.0%) [0]	0 (0.0%) [0]	0 (0.0%) [0]	1 (0.4%) [1]	0 (0.0%) [0]
Productive cough	0 (0.0%) [0]	0 (0.0%) [0]	1 (0.4%) [1]	0 (0.0%) [0]	0 (0.0%) [0]
Skin and subcutaneous tissue disorders	0 (0.0%) [0]	1 (0.4%) [1]	0 (0.0%) [0]	0 (0.0%) [0]	0 (0.0%) [0]
Cold sweat	0 (0.0%) [0]	0 (0.0%) [0]	0 (0.0%) [0]	0 (0.0%) [0]	0 (0.0%) [0]
Hyperhidrosis	0 (0.0%) [0]	0 (0.0%) [0]	0 (0.0%) [0]	0 (0.0%) [0]	0 (0.0%) [0]
Blister	0 (0.0%) [0]	0 (0.0%) [0]	0 (0.0%) [0]	0 (0.0%) [0]	0 (0.0%) [0]
Dermatitis	0 (0.0%) [0]	0 (0.0%) [0]	0 (0.0%) [0]	0 (0.0%) [0]	0 (0.0%) [0]
Dermatitis atopic	0 (0.0%) [0]	1 (0.4%) [1]	0 (0.0%) [0]	0 (0.0%) [0]	0 (0.0%) [0]
Vascular disorders	0 (0.0%) [0]	0 (0.0%) [0]	0 (0.0%) [0]	0 (0.0%) [0]	0 (0.0%) [0]
Hot flush	0 (0.0%) [0]	0 (0.0%) [0]	0 (0.0%) [0]	0 (0.0%) [0]	0 (0.0%) [0]
Pallor	0 (0.0%) [0]	0 (0.0%) [0]	0 (0.0%) [0]	0 (0.0%) [0]	0 (0.0%) [0]
Psychiatric disorders	0 (0.0%) [0]	0 (0.0%) [0]	0 (0.0%) [0]	0 (0.0%) [0]	0 (0.0%) [0]
Anxiety	0 (0.0%) [0]	0 (0.0%) [0]	0 (0.0%) [0]	0 (0.0%) [0]	0 (0.0%) [0]
General disorders and administration site conditions	1 (0.4%) [1]	0 (0.0%) [0]	0 (0.0%) [0]	0 (0.0%) [0]	0 (0.0%) [0]
Chest pain	0 (0.0%) [0]	0 (0.0%) [0]	0 (0.0%) [0]	0 (0.0%) [0]	0 (0.0%) [0]
Unevaluable event	1 (0.4%) [1]	0 (0.0%) [0]	0 (0.0%) [0]	0 (0.0%) [0]	0 (0.0%) [0]
Infections and infestations	0 (0.0%) [0]	1 (0.4%) [1]	1 (0.4%) [2]	3 (1.2%) [3]	1 (0.4%) [1]
Conjunctivitis	0 (0.0%) [0]	0 (0.0%) [0]	1 (0.4%) [1]	0 (0.0%) [0]	0 (0.0%) [0]
Influenza	0 (0.0%) [0]	1 (0.4%) [1]	0 (0.0%) [0]	0 (0.0%) [0]	0 (0.0%) [0]
Upper respiratory tract infection	0 (0.0%) [0]	0 (0.0%) [0]	1 (0.4%) [1]	1 (0.4%) [1]	0 (0.0%) [0]
Respiratory tract infection	0 (0.0%) [0]	0 (0.0%) [0]	0 (0.0%) [0]	1 (0.4%) [1]	0 (0.0%) [0]
COVID-19	0 (0.0%) [0]	0 (0.0%) [0]	0 (0.0%) [0]	1 (0.4%) [1]	1 (0.4%) [1]

Injury, poisoning and procedural complications	0 (0.0%) [0]	0 (0.0%) [0]	0 (0.0%) [0]	0 (0.0%) [0]	0 (0.0%) [0]
Road traffic accident	0 (0.0%) [0]	0 (0.0%) [0]	0 (0.0%) [0]	0 (0.0%) [0]	0 (0.0%) [0]

Virginia Tobacco, N=260; Autumn Tobacco, N=237; Polar Menthol, N=262; Summer Menthol, N=249; Ruby Menthol, N=234.

Note. Participants reporting more than one event in a category are counted only once for that category.

*n (%) is the number and percent of participants with AEs and [E] is the total number of AEs reported, as multiple AEs could occur per session.
