# Supplementary material for: Evaluating the Effect of the JUUL2 System With 5 Flavors on Cigarette Smoking and Tobacco Product Use Behaviors Among Adults Who Smoke Cigarettes: 6-Week Actual Use Study
Source: Interact J Med Res. 2025 Mar 26;14:e60620. doi: 10.2196/60620 (PMC11982753; doi:10.2196/60620)
Supplement: Multimedia Appendix 8 [file ijmr_v14i1e60620_app8.pdf]

Multimedia Appendix 8. Association of JUUL2 Flavor Group and Cigarette Flavor with Past 7-Day and Past 30-Day Switch Rates across Six-Week Actual Use Period

Regressor	Past 7-Day Switching		Past 30-Day Switching	
	OR (95% CI)	*p*-value	OR (95% CI)	*p*-value
JUUL2 Flavor Group (Menthol vs. Tobacco)[a]	1.23 (0.92, 1.64)	0.17	1.36 (1.04, 1.78)	0.03
Cigarette Flavor (Mentholated vs. Nonmentholated)	1.52 (1.14, 2.04)	0.005	1.34 (0.99, 1.81)	0.06
Linear Time Trend[b]	1.09 (1.07, 1.12)	<0.001	—	0.10
JUUL2 Flavor Group × Cigarette Flavor Interaction[c]	—	0.03	—	—
JUUL2 Flavor Group × Time Interaction	—	0.82	—	—
Cigarette Flavor × Time Interaction	—	0.20	—	—
JUUL2 Flavor Group × Cigarette Flavor × Time Interaction	—	0.49	—	—

Note. Abbreviations: OR, odds ratio.

N=6,309 observations (1,159 participants). N=1,078 participants.

[a]Virginia Tobacco and Autumn Tobacco vs. Polar Menthol, Summer Menthol and Ruby Menthol

[b]Time coded as weeks since baseline (continuous variable: 1-6); estimated trend reflects the unit change per week.

[c]Reference group = Tobacco group.
