# Supplementary material for: Evaluating the Effect of the JUUL2 System With 5 Flavors on Cigarette Smoking and Tobacco Product Use Behaviors Among Adults Who Smoke Cigarettes: 6-Week Actual Use Study
Source: Interact J Med Res. 2025 Mar 26;14:e60620. doi: 10.2196/60620 (PMC11982753; doi:10.2196/60620)
Supplement: Multimedia Appendix 9 [file ijmr_v14i1e60620_app9.pdf]

Six-Week Actual Use Study to Evaluate the Effect of the JUUL2 System in Five Flavors on Cigarette Smoking and Tobacco Product Use Behaviors among US Adults who Smoke

Multimedia Appendix 9. Rates of Past 30-Day and Past 7-Day Switching Away from Cigarettes across 6-Week Actual Use Period by JUUL2 Flavor Imputing Missing as Smoking (Intent-to-Treat)

JUUL2 pod Flavor Group	Past 7-Day Switching (ITT)						Past 30-Day Switching (ITT)
	Week 1 Follow-Up N (%)	Week 2 Follow-Up N (%)	Week 3 Follow-Up N (%)	Week 4 Follow-Up N (%)	Week 5 Follow-Up N (%)	Week 6 Follow-Up N (%)	Week 6 Follow-Up N (%) [95% CI]
Virginia Tobacco (N=242)	62 (25.6%)	75 (31.0%)	76 (31.4%)	81 (33.5%)	78 (32.2%)	79 (32.6%)	55 (22.7%) [17.5%, 28.0%]
Polar Menthol (N=239)	73 (30.5%)	87 (36.4%)	83 (34.7%)	92 (38.5%)	88 (36.8%)	96 (40.2%)	70 (29.3%) [23.5%, 35.1%]
Autumn Tobacco (N=219)	73 (33.3%)	72 (32.9%)	82 (37.4%)	82 (37.4%)	72 (32.9%)	73 (33.3%)	58 (26.5%) [20.6%, 32.3%]
Summer Menthol (N=236)	77 (32.6%)	80 (33.9%)	87 (36.9%)	93 (39.4%)	95 (40.3%)	99 (41.9%)	74 (31.4%) [25.4%, 37.3%]
Ruby Menthol (N=224)	76 (33.9%)	73 (32.6%)	91 (40.6%)	98 (43.8%)	85 (37.9%)	95 (42.4%)	69 (30.8%) [24.8%, 37.3%]

Note. Abbreviations: ITT, Intent-to-treat. In ITT analyses all missing data were coded as smoking (i.e., not switched).
