# Supplementary material for: Evaluating the Effect of the JUUL2 System With 5 Flavors on Cigarette Smoking and Tobacco Product Use Behaviors Among Adults Who Smoke Cigarettes: 6-Week Actual Use Study
Source: Interact J Med Res. 2025 Mar 26;14:e60620. doi: 10.2196/60620 (PMC11982753; doi:10.2196/60620)
Supplement: Multimedia Appendix 10 [file ijmr_v14i1e60620_app10.pdf]

Multimedia Appendix 10. Sociodemographic and Tobacco Use Characteristics of Participants who Smoked Nonmentholated Cigarettes by JUUL2 Flavor Selection

Sample Characteristics	N (%) or Mean (*SD*)		*p*-value
	Tobacco-Flavored JUUL2 (N=158)	Menthol-Flavored JUUL2 (N=151)	
**Sociodemographic Characteristics**			
Age, yr, *Mean* (*SD*)	40.88 (11.47)	39.88 (11.17)	0.439
Sex			
Male	73 (46.2%)	66 (43.7%)	0.689
Female	85 (53.8%)	84 (55.6%)	
Other	0 (0.0%)	0 (0.0%)	
Prefer not to answer	0 (0.0%)	1 (0.7%)	
Race/Ethnicity			
Non-Hispanic White	122 (77.2%)	109 (72.2%)	0.370
Non-Hispanic Black	11 (7.0%)	19 (12.6%)	
Non-Hispanic Other Race	8 (5.1%)	4 (2.6%)	
Hispanic Ethnicity	15 (9.5%)	16 (10.6%)	
Unknown	2 (1.3%)	3 (2.0%)	
Marital Status			
Married	57 (36.1%)	44 (29.1%)	0.524
Living with Partner	26 (16.5%)	34 (22.5%)	
Divorced, Separated or Widowed	28 (17.7%)	27 (17.9%)	
Never Married	44 (27.8%)	41 (27.2%)	
Prefer not to say	3 (1.9%)	5 (3.3%)	
Annual Household Income			
<$50,000	91 (57.6%)	92 (60.9%)	0.762
$50,000-$99,999	50 (31.6%)	42 (27.8%)	
$100,000 or more	17 (10.8%)	17 (11.3%)	
Highest Level of Education			
High school graduate or less	62 (39.2%)	67 (44.4%)	0.228
Some college or trade school	55 (34.8%)	57 (37.7%)	
College graduate or more education	41 (25.9%)	27 (17.9%)	
Employment Status			
Full time	89 (56.3%)	95 (62.9%)	0.334
Part time	21 (13.3%)	13 (8.6%)	
Other	48 (30.4%)	43 (28.5%)	
**Smoking Characteristics**			
No. Cigarettes Smoked per Smoking Day, *Mean* (*SD*)	15.85 (8.27)	14.74 (7.38)	0.214
Duration of Smoking, yr *Mean* (*SD*)	19.08 (10.74)	17.93 (10.62)	0.348
Age started smoking, yr, *Mean* (*SD*)	18.04 (6.46)	18.10 (4.08)	0.928
Cigarette Dependence[a] *Mean* (*SD*)	3.64 (0.81)	3.62 (0.79)	0.818
Plan to Quit Smoking in Next 30 Days	4 (2.5%)	3 (2.0%)	1.000
Ever Plan to Quit Smoking	55 (34.8%)	54 (35.8%)	0.861
**ENDS Use Characteristics**			
Ever used ENDS	107 (67.7%)	97 (64.2%)	0.518
Age First Used ENDS, yr, *Mean* (*SD*)	34.07 (12.61)	32.26 (12.14)	0.299
Ever used ENDS Fairly Regularly	66 (61.7%)	57 (58.8%)	0.670
Used ENDS in Past 30 Days	53 (49.5%)	55 (56.7%)	0.306
No. Days Used ENDS in P30D, *Mean* (*SD*)	16.83 (9.87)	14.91 (9.57)	0.307
ENDS Dependence,[a] *Mean* (*SD*)	2.87 (0.84)	2.68 (0.91)	0.259

Primary ENDS Flavor[b]			
Tobacco	17 (32.1%)	13 (23.6%)	
Menthol	6 (11.3%)	6 (10.9%)	
Mint	1 (1.9%)	2 (3.6%)	
Fruit	21 (39.6%)	25 (45.5%)	0.937
Dessert/Candy	7 (13.2%)	7 (12.7%)	
Spice/Clove	0 (0.0%)	1 (1.8%)	
Some other flavor	1 (1.9%)	1 (1.8%)	
Primary ENDS Device Type[b]			
Pod-based	19 (35.8%)	18 (32.7%)	
Disposable	23 (43.4%)	26 (47.3%)	0.973
Tank	9 (17.0%)	8 (14.5%)	
Mod	0 (0.0%)	1 (1.8%)	

Note. Values represent N (%) or Mean (SD) unless otherwise noted. Denominators may be less than totals in column heads.

[a]Tobacco Dependence Index in PATH adult survey (Range: 1-5; higher scores indicate greater dependence).

[b]Participants selected the single flavor, nicotine concentration or ENDS device they used most often.

[c]Includes smokeless tobacco and tobacco-free “modern” oral nicotine pouches.
