# Supplementary material for: Evaluating the Effect of the JUUL2 System With 5 Flavors on Cigarette Smoking and Tobacco Product Use Behaviors Among Adults Who Smoke Cigarettes: 6-Week Actual Use Study
Source: Interact J Med Res. 2025 Mar 26;14:e60620. doi: 10.2196/60620 (PMC11982753; doi:10.2196/60620)
Supplement: Multimedia Appendix 11 [file ijmr_v14i1e60620_app11.pdf]

Six-Week Actual Use Study to Evaluate the Effect of the JUUL2 System in Five Flavors on Cigarette Smoking and Tobacco Product Use Behaviors among US Adults who Smoke

Multimedia Appendix 11. Association of Past 30-Day ENDS Use at Baseline with Past 7-Day Switch Rates across Six-Week Actual Use Period

Regressor	Past 7-Day Switching	
	OR (95% CI)	*p*-value
Past 30-Day ENDS Use at Baseline (Yes vs. No)	0.61 (0.49, 0.75)	<0.001
Linear Time Trend[a]	1.09 (1.07, 1.11)	<0.001
Past 30-Day ENDS Use at Baseline[b] × Time Interaction	—	0.576

Note. Abbreviations: OR, odds ratio.

N=6,315 observations (1,160 participants).

[a]Time coded as weeks since baseline (continuous variable: 1-6); estimated trend reflects the unit change per week.

[b]Reference group = No Past 30-Day ENDS Use at Baseline.
