# Supplementary material for: Evaluating the Effect of the JUUL2 System With 5 Flavors on Cigarette Smoking and Tobacco Product Use Behaviors Among Adults Who Smoke Cigarettes: 6-Week Actual Use Study
Source: Interact J Med Res. 2025 Mar 26;14:e60620. doi: 10.2196/60620 (PMC11982753; doi:10.2196/60620)
Supplement: Multimedia Appendix 12 [file ijmr_v14i1e60620_app12.pdf]

Six-Week Actual Use Study to Evaluate the Effect of the JUUL2 System in Five Flavors on Cigarette Smoking and Tobacco Product Use Behaviors among US Adults who Smoke

Multimedia Appendix 12. Change in Daily Cigarette Consumption from Baseline to Week 6 among JUUL2 Flavor Groups

JUUL2 Flavor	Cigarette Smoking and Reduction	Week 1 Survey (N=128-161)	Week 2 Survey (N=133-159)	Week 3 Survey (N=114-147)	Week 4 Survey (N=108-139)	Week 5 Survey (N=111-137)	Week 6 Survey (N=106-128)
Virginia Tobacco	Baseline Cigarette Consumption, Mean (*SD*)	13.61 (8.54)	13.40 (8.32)	13.65 (8.47)	14.24 (8.53)	13.96 (8.49)	14.17 (8.62)
	Cigarette Consumption at Week 6, Mean (*SD*)	7.37 (6.04)	7.30 (5.94)	7.12 (6.27)	8.23 (8.82)	8.20 (7.47)	8.60 (8.87)
	≥50% Reduction from baseline, N (%)	90 (55.9%)	86 (54.1%)	88 (59.9%)	71 (51.1%)	68 (49.6%)	68 (53.1%)
	Percent Reduction, Median (IQR)	50.0 (69.6)	50.0 (64.3)	57.1 (63.3)	50.0 (60.7)	46.4 (77.1)	50.0 (60.0)
Autumn Tobacco	Baseline Cigarette Consumption, Mean (*SD*)	16.62 (10.53)	17.09 (11.62)	17.09 (10.78)	16.70 (10.15)	17.22 (10.67)	17.03 (10.69)
	Cigarette Consumption at Week 6, Mean (*SD*)	8.28 (8.79)	9.04 (9.68)	8.43 (8.54)	8.24 (8.48)	8.94 (9.30)	7.95 (8.47)
	≥50% Reduction from baseline, N (%)	76 (58.5)	69 (51.9)	66 (56.4)	71 (59.2)	71 (60.2)	73 (62.9)
	Percent Reduction, Median (IQR)	56.1 (65.5%)	50.0 (64.3%)	50.0 (61.0%)	57.7 (56.9%)	60.0 (61.0%)	63.4 (58.4%)
Polar Menthol	Baseline Cigarette Consumption, Mean (*SD*)	12.39 (6.41)	12.83 (6.46)	12.74 (6.59)	12.75 (6.51)	12.93 (6.50)	12.97 (6.42)
	Cigarette Consumption at Week 6, (Mean (*SD*)	7.86 (7.85)	6.97 (6.23)	7.50 (7.99)	6.97 (6.27)	6.96 (6.95)	7.91 (7.78)
	≥50% Reduction from baseline, N (%)	75 (49.7%)	71 (52.2%)	69 (53.1%)	69 (54.8%)	68 (55.7%)	59 (50.9%)
	Percent Reduction, Median (IQR)	46.7 (80.0%)	50.0 (69.3%)	50.0 (75.7%)	57.6 (82.9%)	57.1 (77.1%)	50.0 (81.9%)
Summer Menthol	Baseline Cigarette Consumption, Mean (*SD*)	14.16 (8.34)	14.36 (8.95)	14.36 (8.51)	14.40 (8.64)	14.21 (8.39)	14.36 (8.57)
	Cigarette Consumption at Week 6, Mean (*SD*)	6.00 (5.75)	6.14 (6.01)	5.83 (5.73)	6.11 (6.00)	6.00 (6.54)	6.23 (6.14)
	≥50% Reduction from baseline, N (%)	82 (59.4%)	88 (64.2%)	84 (68.3%)	78 (64.5%)	79 (68.7%)	69 (61.6%)
	Percent Reduction, Median (IQR)	61.4 (60.7)	62.5 (52.4)	60.0 (48.2)	60.0 (49.1)	70.0 (44.8)	63.5 (55.7)
Ruby Menthol	Baseline Cigarette Consumption, Mean (*SD*)	15.07 (7.63)	15.06 (7.66)	15.40 (8.11)	15.25 (8.09)	15.37 (7.92)	15.10 (7.69)
	Cigarette Consumption at Week 6, Mean (*SD*)	7.11 (6.18)	7.42 (7.81)	7.79 (8.35)	7.80 (7.67)	7.78 (7.45)	8.18 (7.50)
	≥50% Reduction from baseline, N (%)	78 (60.9)	84 (61.3%)	70 (61.4%)	62 (57.4%)	71 (64.0%)	59 (55.7%)
	Percent Reduction, Median (IQR)	57.7 (65.2)	64.3 (55.0)	57.3 (51.4)	54.4 (53.5)	61.9 (58.3)	51.0 (77.4)
