# Supplementary material for: Evaluating the Effect of the JUUL2 System With 5 Flavors on Cigarette Smoking and Tobacco Product Use Behaviors Among Adults Who Smoke Cigarettes: 6-Week Actual Use Study
Source: Interact J Med Res. 2025 Mar 26;14:e60620. doi: 10.2196/60620 (PMC11982753; doi:10.2196/60620)
Supplement: Multimedia Appendix 13 [file ijmr_v14i1e60620_app13.pdf]

Multimedia Appendix 13. Patterns of Past 7-Day JUUL2 Product Use across Six-Week Actual Use Period among JUUL2 Flavor Groups

JUUL2 Flavor	Patterns of JUUL2 Product Use	Week 1 Follow-Up	Week 2 Follow-Up	Week 3 Follow-Up	Week 4 Follow-Up	Week 5 Follow-Up	Week 6 Follow-Up
Virginia Tobacco	N	222	234	223	220	215	207
	Past 7 Day JUUL2 Use, n (%)	222 (100.0%)	233 (99.6%)	222 (99.6%)	218 (99.1%)	215 (100.0%)	207 (100.0%)
	No. Days Used JUUL2						
	Mean (*SD*)	6.28 (1.57)	6.20 (1.63)	6.13 (1.65)	6.12 (1.63)	6.07 (1.68)	6.12 (1.66)
	Median (IQR)	7 (0)	7 (1)	7 (1)	7 (1)	7 (1)	7 (1)
	No. Times Used JUUL2 per Day						
	Mean (*SD*)	14.38 (17.07)	14.50 (18.61)	15.83 (20.92)	16.54 (22.59)	15.46 (20.16)	15.75 (21.09)
	Median (IQR)	9 (13)	9 (10)	7 (15)	8 (11)	8 (13)	8 (11)
	No. JUUL2 Puffs/Day						
	Mean (*SD*)	25.47 (26.97)	26.97 (16.00)	25.28 (26.87)	25.17 (27.95)	25.63 (28.09)	23.89 (26.90)
	Median (IQR)	16 (26)	15 (28)	15 (24)	14 (24)	13 (34)	12 (24)
Autumn Tobacco	N	204	206	199	203	191	190
	Past 7 Day JUUL2 Use, n (%)	203 (99.5%)	205 (99.5%)	198 (99.5%)	202 (99.5%)	190 (99.5%)	188 (98.9%)
	No. Days Used JUUL2						
	Mean (*SD*)	6.54 (1.10)	6.42 (1.26)	6.48 (1.19)	6.47 (1.17)	6.51 (1.21)	6.44 (1.27)
	Median (IQR)	7 (0)	7 (0)	7 (0)	7 (0)	7 (0)	7 (0)
	No. Times Used JUUL2 per Day						
	Mean (*SD*)	15.74 (15.70)	15.87 (16.63)	15.81 (16.31)	15.68 (17.54)	17.53 (20.35)	17.89 (20.64)
	Median (IQR)	10 (14)	10 (14)	10 (14)	10 (13)	10 (15)	10 (14)
	No. JUUL2 Puffs/Day						
	Mean (*SD*)	25.73 (23.44)	23.44 (20.00)	25.27 (25.09)	23.89 (24.91)	26.16 (27.85)	25.51 (28.17)
	Median (IQR)	20 (26)	15 (22)	15 (23)	14 (23)	15 (33)	12 (27)
Polar Menthol	N	222	222	213	218	210	212
	Past 7 Day JUUL2 Use, n (%)	222 (100.0%)	222 (100.0%)	212 (99.5%)	213 (97.7%)	209 (99.5%)	211 (99.5%)
	No. Days Used JUUL2						
	Mean (*SD*)	6.13 (1.65)	6.01 (1.74)	5.92 (1.79)	6.01 (1.73)	6.00 (1.74)	5.98 (1.76)
	Median (IQR)	7 (1)	7 (2)	7 (2)	7 (1)	7 (2)	7 (2)
	No. Times Used JUUL2 per Day						

	Mean (*SD*)	17.88 (21.90)	19.05 (24.23)	18.51 (23.33)	17.79 (21.18)	19.01 (23.30)	18.84 (22.85)
	Median (IQR)	10 (15)	10 (15)	9 (16.5)	10 (15)	10 (15)	10 (15)
	No. JUUL2 Puffs/Day						
	Mean (*SD*)	27.55 (28.69)	28.69 (17.00)	28.31 (29.40)	31.38 (30.57)	29.56 (30.38)	29.46 (30.48)
	Median (IQR)	17 (32)	18 (35)	17.5 (31)	20 (42)	16 (33)	17 (34)
Summer Menthol	N	215	217	210	214	210	211
	Past 7 Day JUUL2 Use, n (%)	215 (100.0%)	216 (99.5%)	210 (100.0%)	214 (100.0%)	209 (99.5%)	210 (99.5%)
	No. Days Used JUUL2						
	Mean (*SD*)	6.60 (1.00)	6.61 (0.98)	6.51 (1.07)	6.44 (1.17)	6.50 (1.14)	6.40 (1.29)
	Median (IQR)	7 (0)	7 (0)	7 (0)	7 (0)	7 (0)	7 (0)
	No. Times Used JUUL2 per Day						
	Mean (*SD*)	18.66 (19.93)	19.24 (21.88)	19.29 (23.02)	19.39 (22.58)	21.09 (23.93)	20.27 (23.83)
	Median (IQR)	10 (19)	10 (13)	10 (15)	10 (14)	10 (19)	10 (18)
	No. JUUL2 Puffs/Day						
	Mean (*SD*)	28.41 (24.88)	24.88 (20.00)	27.83 (27.54)	28.32 (27.15)	28.55 (27.75)	30.20 (28.94)
	Median (IQR)	20 (31)	20 (29)	19 (33)	16 (36)	18 (32)	20 (34)
Ruby Menthol	N	204	210	204	206	196	201
	Past 7 Day JUUL2 Use, n (%)	204 (100.0%)	210 (100.0%)	204 (100.0%)	203 (98.5%)	192 (98.0%)	198 (98.5%)
	No. Days Used JUUL2						
	Mean (*SD*)	6.61 (0.99)	6.50 (1.10)	6.52 (1.14)	6.37 (1.34)	6.43 (1.27)	6.27 (1.40)
	Median (IQR)	7 (0)	7 (0)	7 (0)	7 (0)	7 (0)	7 (1)
	No. Times Used JUUL2 per Day						
	Mean (*SD*)	18.38 (20.29)	20.40 (22.43)	19.58 (22.63)	21.35 (25.04)	19.78 (23.49)	20.75 (24.35)
	Median (IQR)	10 (14)	10 (18)	10 (19)	10 (20)	10 (14)	10 (20)
	No. JUUL2 Puffs/Day						
	Mean (*SD*)	28.79 (26.57)	26.57 (20.00)	29.61 (29.10)	30.27 (29.79)	30.71 (30.48)	30.61 (31.13)
	Median (IQR)	20 (30)	19.5 (32)	16 (32)	16 (35)	20 (36)	15 (35)

Note. Abbreviations: IQR, interquartile range.
All items assessed JUUL2 product use in past 7 days.
