# Supplementary material for: Evaluating the Effect of the JUUL2 System With 5 Flavors on Cigarette Smoking and Tobacco Product Use Behaviors Among Adults Who Smoke Cigarettes: 6-Week Actual Use Study
Source: Interact J Med Res. 2025 Mar 26;14:e60620. doi: 10.2196/60620 (PMC11982753; doi:10.2196/60620)
Supplement: Multimedia Appendix 14 [file ijmr_v14i1e60620_app14.pdf]

Multimedia Appendix 14. Temporal Trends in Daily JUUL2 Product Use across Six-Week Actual Use Period among JUUL2 Flavor Groups

Dependent Variable	Linear Time Trend	
	B (95% CI)	*p*-value
**Number of Times Used JUUL2 per Day**		
Virginia Tobacco	0.24 (-0.09, 0.57)	0.15
Polar Menthol	0.21 (-0.16, 0.59)	0.27
Autumn Tobacco	0.48 (0.17, 0.79)	<0.01
Summer Menthol	0.45 (0.10, 0.80)	0.01
Ruby Menthol	0.54 (0.12, 0.96)	0.01
**Number of JUUL2 Puffs per Day**		
Virginia Tobacco	-0.39 (-0.85, 0.07)	0.10
Polar Menthol	0.53 (0.05, 1.02)	0.03
Autumn Tobacco	0.11 (-0.37, 0.60)	0.64
Summer Menthol	0.43 (-0.03, 0.88)	0.07
Ruby Menthol	0.60 (0.06, 1.15)	0.03

Note. Abbreviations: B, unstandardized coefficient from mixed effects model.
Time per Day: N=6,277 observations (1,156 participants).
Puffs per Day: N=6,276 observations (1,156 participants).
