# Supplementary material for: Evaluating the Effect of the JUUL2 System With 5 Flavors on Cigarette Smoking and Tobacco Product Use Behaviors Among Adults Who Smoke Cigarettes: 6-Week Actual Use Study
Source: Interact J Med Res. 2025 Mar 26;14:e60620. doi: 10.2196/60620 (PMC11982753; doi:10.2196/60620)
Supplement: Multimedia Appendix 15 [file ijmr_v14i1e60620_app15.pdf]

Multimedia Appendix 15. Subjective Responses to JUUL2 Products at Week 6 Survey among JUUL2 Flavor Groups

Modified Product Evaluation Scale Subscale or Individual Item	Virginia Tobacco	Polar Menthol	Autumn Tobacco	Summer Menthol	Ruby Menthol
N	207	210	187	210	198
mPES Satisfaction Subscale	5.08 (1.40)	5.22 (1.27)	5.18 (1.34)	5.24 (1.34)	5.11 (1.31)
mPES Psychological Reward Subscale	4.26 (1.50)	4.30 (1.39)	4.28 (1.49)	4.29 (1.54)	3.98 (1.53)
mPES Aversion Subscale	1.62 (0.93)	1.55 (0.88)	1.71 (1.15)	1.69 (1.03)	1.72 (1.01)
mPES Relief Subscale	4.71 (1.29)	4.75 (1.26)	4.77 (1.19)	4.82 (1.14)	4.65 (1.26)

Note. Values represent mean (*SD*).
Items were rated on a seven-point response scale, ranging from 1 (“Not at all”) to 7 (“Extremely”).
