# Supplementary material for: Evaluating the Effect of the JUUL2 System With 5 Flavors on Cigarette Smoking and Tobacco Product Use Behaviors Among Adults Who Smoke Cigarettes: 6-Week Actual Use Study
Source: Interact J Med Res. 2025 Mar 26;14:e60620. doi: 10.2196/60620 (PMC11982753; doi:10.2196/60620)
Supplement: Multimedia Appendix 16 [file ijmr_v14i1e60620_app16.pdf]

Six-Week Actual Use Study to Evaluate the Effect of the JUUL2 System in Five Flavors on Cigarette Smoking and Tobacco Product Use Behaviors among US Adults who Smoke

Multimedia Appendix 16. Adverse Events – Traditional and Complex Flavors Trial Week

Adverse Events (AEs)	Traditional Flavors n (%) [E]*	Complex Flavors n (%) [E]*
Total Participants Reporting at Least One AE	1 (0.2%) [2]	4 (0.4%) [7]
Serious AEs	0 (0.0%) [0]	0 (0.0%) [0]
Death	0 (0.0%) [0]	0 (0.0%) [0]
Life Threatening	0 (0.0%) [0]	0 (0.0%) [0]
Hospitalization	0 (0.0%) [0]	0 (0.0%) [0]
Disabling	0 (0.0%) [0]	0 (0.0%) [0]
Birth Defect	0 (0.0%) [0]	0 (0.0%) [0]
Medically Important	0 (0.0%) [0]	0 (0.0%) [0]
Maximum Intensity		
Severe	0 (0.0%) [0]	0 (0.0%) [0]
Moderate	1 (0.2%) [2]	0 (0.0%) [0]
Mild	0 (0.0%) [0]	4 (0.4%) [7]
Unknown	0 (0.0%) [0]	0 (0.0%) [0]
Worst Relationship		
Related	0 (0.0%) [0]	0 (0.0%) [0]
Probably Related	1 (0.2%) [2]	2 (0.2%) [5]
Possibly Related	0 (0.0%) [0]	1 (0.1%) [1]
Unlikely Related	0 (0.0%) [0]	0 (0.0%) [0]
Not Related	0 (0.0%) [0]	1 (0.1%) [1]
Outcome of AE		
Fatal	0 (0.0%) [0]	0 (0.0%) [0]
Worse	0 (0.0%) [0]	0 (0.0%) [0]
Unchanged	0 (0.0%) [0]	0 (0.0%) [0]
Improved	0 (0.0%) [0]	0 (0.0%) [0]
Resolved	1 (0.2%) [2]	4 (0.4%) [7]
Unknown	0 (0.0%) [0]	0 (0.0%) [0]
MedDRA System Organ Class<br>Preferred Term		
Nervous system disorders	0 (0.0%) [0]	0 (0.0%) [0]
Dizziness	0 (0.0%) [0]	0 (0.0%) [0]
Headache	0 (0.0%) [0]	0 (0.0%) [0]
Somnolence	0 (0.0%) [0]	0 (0.0%) [0]
Syncope	0 (0.0%) [0]	0 (0.0%) [0]
Hypoaesthesia	0 (0.0%) [0]	0 (0.0%) [0]
Gastrointestinal disorders	0 (0.0%) [0]	0 (0.0%) [0]
Abdominal pain upper	0 (0.0%) [0]	0 (0.0%) [0]
Constipation	0 (0.0%) [0]	0 (0.0%) [0]
Diarrhea	0 (0.0%) [0]	0 (0.0%) [0]
Nausea	0 (0.0%) [0]	0 (0.0%) [0]
Vomiting	0 (0.0%) [0]	0 (0.0%) [0]
Respiratory, thoracic, and mediastinal disorders	1 (0.2%) [1]	1 (0.1%) [4]
Cough	0 (0.0%) [0]	1 (0.1%) [1]
Throat irritation	0 (0.0%) [0]	1 (0.1%) [1]
Dysphonia	0 (0.0%) [0]	1 (0.1%) [1]
Dyspnoea	1 (0.2%) [1]	0 (0.0%) [0]
Haemoptysis	0 (0.0%) [0]	1 (0.1%) [1]

Nasal congestion	0 (0.0%) [0]	0 (0.0%) [0]
Productive cough	0 (0.0%) [0]	0 (0.0%) [0]
Skin and subcutaneous tissue disorders	0 (0.0%) [0]	1 (0.1%) [1]
Cold sweat	0 (0.0%) [0]	0 (0.0%) [0]
Hyperhidrosis	0 (0.0%) [0]	0 (0.0%) [0]
Blister	0 (0.0%) [0]	0 (0.0%) [0]
Dermatitis	0 (0.0%) [0]	1 (0.1%) [1]
Dermatitis atopic	0 (0.0%) [0]	0 (0.0%) [0]
Vascular disorders	0 (0.0%) [0]	0 (0.0%) [0]
Hot flush	0 (0.0%) [0]	0 (0.0%) [0]
Pallor	0 (0.0%) [0]	0 (0.0%) [0]
Psychiatric disorders	0 (0.0%) [0]	0 (0.0%) [0]
Anxiety	0 (0.0%) [0]	0 (0.0%) [0]
General disorders and administration site conditions	1 (0.2%) [1]	1 (0.1%) [1]
Chest pain	1 (0.2%) [1]	1 (0.1%) [1]
Unevaluable event	0 (0.0%) [0]	0 (0.0%) [0]
Infections and infestations	0 (0.0%) [0]	0 (0.0%) [0]
Conjunctivitis	0 (0.0%) [0]	0 (0.0%) [0]
Influenza	0 (0.0%) [0]	0 (0.0%) [0]
Upper respiratory tract infection	0 (0.0%) [0]	0 (0.0%) [0]
Respiratory tract infection	0 (0.0%) [0]	0 (0.0%) [0]
COVID-19	0 (0.0%) [0]	0 (0.0%) [0]
Injury, poisoning and procedural complications	0 (0.0%) [0]	1 (0.1%) [1]
Road traffic accident	0 (0.0%) [0]	1 (0.1%) [1]

Traditional Flavors, N=648; Complex Flavors, N=910.

Note. Participants reporting more than one event in a category are counted only once for that category.

*n (%) is the number and percent of participants with AEs and [E] is the total number of AEs reported, as multiple AEs could occur per session.
