# Supplementary material for: Evaluating the Effect of the JUUL2 System With 5 Flavors on Cigarette Smoking and Tobacco Product Use Behaviors Among Adults Who Smoke Cigarettes: 6-Week Actual Use Study
Source: Interact J Med Res. 2025 Mar 26;14:e60620. doi: 10.2196/60620 (PMC11982753; doi:10.2196/60620)
Supplement: Multimedia Appendix 17 [file ijmr_v14i1e60620_app17.pdf]

Six-Week Actual Use Study to Evaluate the Effect of the JUUL2 System in Five Flavors on Cigarette Smoking and Tobacco Product Use Behaviors among US Adults who Smoke

Multimedia Appendix 17. Adverse Events over Six-Week Actual Use Period among JUUL2 Flavor Groups

Adverse Events (AEs)	Virginia Tobacco n (%) [E]*	Autumn Tobacco n (%) [E]*	Polar Menthol n (%) [E]*	Summer Menthol n (%) [E]*	Ruby Menthol n (%) [E]*
Total Participants Reporting at Least One AE	1 (0.4%) [1]	2 (0.8%) [2]	2 (0.8%) [4]	6 (2.4%) [6]	2 (0.9%) [2]
Serious AEs	0 (0.0%) [0]	0 (0.0%) [0]	0 (0.0%) [0]	0 (0.0%) [0]	0 (0.0%) [0]
Death	0 (0.0%) [0]	0 (0.0%) [0]	0 (0.0%) [0]	0 (0.0%) [0]	0 (0.0%) [0]
Life Threatening	0 (0.0%) [0]	0 (0.0%) [0]	0 (0.0%) [0]	0 (0.0%) [0]	0 (0.0%) [0]
Hospitalization	0 (0.0%) [0]	0 (0.0%) [0]	0 (0.0%) [0]	0 (0.0%) [0]	0 (0.0%) [0]
Disabling	0 (0.0%) [0]	0 (0.0%) [0]	0 (0.0%) [0]	0 (0.0%) [0]	0 (0.0%) [0]
Birth Defect	0 (0.0%) [0]	0 (0.0%) [0]	0 (0.0%) [0]	0 (0.0%) [0]	0 (0.0%) [0]
Medically Important	0 (0.0%) [0]	0 (0.0%) [0]	0 (0.0%) [0]	0 (0.0%) [0]	0 (0.0%) [0]
Maximum Intensity					
Severe	0 (0.0%) [0]	0 (0.0%) [0]	0 (0.0%) [0]	0 (0.0%) [0]	0 (0.0%) [0]
Moderate	0 (0.0%) [0]	1 (0.4%) [1]	1 (0.4%) [1]	1 (0.4%) [1]	1 (0.4%) [1]
Mild	0 (0.0%) [0]	1 (0.4%) [1]	2 (0.8%) [3]	5 (2.0%) [5]	1 (0.4%) [1]
Unknown	1 (0.4%) [1]	0 (0.0%) [0]	0 (0.0%) [0]	0 (0.0%) [0]	0 (0.0%) [0]
Worst Relationship					
Related	0 (0.0%) [0]	0 (0.0%) [0]	0 (0.0%) [0]	0 (0.0%) [0]	0 (0.0%) [0]
Probably Related	0 (0.0%) [0]	0 (0.0%) [0]	0 (0.0%) [0]	1 (0.4%) [1]	0 (0.0%) [0]
Possibly Related	0 (0.0%) [0]	0 (0.0%) [0]	1 (0.4%) [2]	0 (0.0%) [0]	1 (0.4%) [1]
Unlikely Related	0 (0.0%) [0]	1 (0.4%) [1]	0 (0.0%) [0]	3 (1.2%) [3]	0 (0.0%) [0]
Not Related	1 (0.4%) [1]	1 (0.4%) [1]	1 (0.4%) [2]	2 (0.8%) [2]	1 (0.4%) [1]
Outcome of AE					
Fatal	0 (0.0%) [0]	0 (0.0%) [0]	0 (0.0%) [0]	0 (0.0%) [0]	0 (0.0%) [0]
Worse	0 (0.0%) [0]	0 (0.0%) [0]	0 (0.0%) [0]	0 (0.0%) [0]	0 (0.0%) [0]
Unchanged	0 (0.0%) [0]	0 (0.0%) [0]	0 (0.0%) [0]	0 (0.0%) [0]	0 (0.0%) [0]
Improved	0 (0.0%) [0]	0 (0.0%) [0]	0 (0.0%) [0]	0 (0.0%) [0]	0 (0.0%) [0]
Resolved	0 (0.0%) [0]	2 (0.8%) [2]	2 (0.8%) [4]	5 (2.0%) [5]	2 (0.9%) [2]
Unknown	1 (0.4%) [1]	0 (0.0%) [0]	0 (0.0%) [0]	1 (0.4%) [1]	0 (0.0%) [0]
MedDRA System Organ Class<br>Preferred Term					
Nervous system disorders	0 (0.0%) [0]	0 (0.0%) [0]	0 (0.0%) [0]	1 (0.4%) [1]	0 (0.0%) [0]
Dizziness	0 (0.0%) [0]	0 (0.0%) [0]	0 (0.0%) [0]	0 (0.0%) [0]	0 (0.0%) [0]
Headache	0 (0.0%) [0]	0 (0.0%) [0]	0 (0.0%) [0]	0 (0.0%) [0]	0 (0.0%) [0]
Somnolence	0 (0.0%) [0]	0 (0.0%) [0]	0 (0.0%) [0]	0 (0.0%) [0]	0 (0.0%) [0]

Syncope	0 (0.0%) [0]	0 (0.0%) [0]	0 (0.0%) [0]	0 (0.0%) [0]	0 (0.0%) [0]
Hypoaesthesia	0 (0.0%) [0]	0 (0.0%) [0]	0 (0.0%) [0]	1 (0.4%) [1]	0 (0.0%) [0]
Gastrointestinal disorders	0 (0.0%) [0]	0 (0.0%) [0]	0 (0.0%) [0]	0 (0.0%) [0]	0 (0.0%) [0]
Abdominal pain upper	0 (0.0%) [0]	0 (0.0%) [0]	0 (0.0%) [0]	0 (0.0%) [0]	0 (0.0%) [0]
Constipation	0 (0.0%) [0]	0 (0.0%) [0]	0 (0.0%) [0]	0 (0.0%) [0]	0 (0.0%) [0]
Diarrhea	0 (0.0%) [0]	0 (0.0%) [0]	0 (0.0%) [0]	0 (0.0%) [0]	0 (0.0%) [0]
Nausea	0 (0.0%) [0]	0 (0.0%) [0]	0 (0.0%) [0]	0 (0.0%) [0]	0 (0.0%) [0]
Vomiting	0 (0.0%) [0]	0 (0.0%) [0]	0 (0.0%) [0]	0 (0.0%) [0]	0 (0.0%) [0]
Respiratory, thoracic, and mediastinal disorders	0 (0.0%) [0]	0 (0.0%) [0]	1 (0.4%) [2]	2 (0.8%) [2]	1 (0.4%) [1]
Cough	0 (0.0%) [0]	0 (0.0%) [0]	0 (0.0%) [0]	0 (0.0%) [0]	1 (0.4%) [1]
Throat irritation	0 (0.0%) [0]	0 (0.0%) [0]	0 (0.0%) [0]	1 (0.4%) [1]	0 (0.0%) [0]
Dysphonia	0 (0.0%) [0]	0 (0.0%) [0]	1 (0.4%) [1]	0 (0.0%) [0]	0 (0.0%) [0]
Dyspnoea	0 (0.0%) [0]	0 (0.0%) [0]	0 (0.0%) [0]	0 (0.0%) [0]	0 (0.0%) [0]
Haemoptysis	0 (0.0%) [0]	0 (0.0%) [0]	0 (0.0%) [0]	0 (0.0%) [0]	0 (0.0%) [0]
Nasal congestion	0 (0.0%) [0]	0 (0.0%) [0]	0 (0.0%) [0]	1 (0.4%) [1]	0 (0.0%) [0]
Productive cough	0 (0.0%) [0]	0 (0.0%) [0]	1 (0.4%) [1]	0 (0.0%) [0]	0 (0.0%) [0]
Skin and subcutaneous tissue disorders	0 (0.0%) [0]	1 (0.4%) [1]	0 (0.0%) [0]	0 (0.0%) [0]	0 (0.0%) [0]
Cold sweat	0 (0.0%) [0]	0 (0.0%) [0]	0 (0.0%) [0]	0 (0.0%) [0]	0 (0.0%) [0]
Hyperhidrosis	0 (0.0%) [0]	0 (0.0%) [0]	0 (0.0%) [0]	0 (0.0%) [0]	0 (0.0%) [0]
Blister	0 (0.0%) [0]	0 (0.0%) [0]	0 (0.0%) [0]	0 (0.0%) [0]	0 (0.0%) [0]
Dermatitis	0 (0.0%) [0]	0 (0.0%) [0]	0 (0.0%) [0]	0 (0.0%) [0]	0 (0.0%) [0]
Dermatitis atopic	0 (0.0%) [0]	1 (0.4%) [1]	0 (0.0%) [0]	0 (0.0%) [0]	0 (0.0%) [0]
Vascular disorders	0 (0.0%) [0]	0 (0.0%) [0]	0 (0.0%) [0]	0 (0.0%) [0]	0 (0.0%) [0]
Hot flush	0 (0.0%) [0]	0 (0.0%) [0]	0 (0.0%) [0]	0 (0.0%) [0]	0 (0.0%) [0]
Pallor	0 (0.0%) [0]	0 (0.0%) [0]	0 (0.0%) [0]	0 (0.0%) [0]	0 (0.0%) [0]
Psychiatric disorders	0 (0.0%) [0]	0 (0.0%) [0]	0 (0.0%) [0]	0 (0.0%) [0]	0 (0.0%) [0]
Anxiety	0 (0.0%) [0]	0 (0.0%) [0]	0 (0.0%) [0]	0 (0.0%) [0]	0 (0.0%) [0]
General disorders and administration site conditions	1 (0.4%) [1]	0 (0.0%) [0]	0 (0.0%) [0]	0 (0.0%) [0]	0 (0.0%) [0]
Chest pain	0 (0.0%) [0]	0 (0.0%) [0]	0 (0.0%) [0]	0 (0.0%) [0]	0 (0.0%) [0]
Unevaluable event	1 (0.4%) [1]	0 (0.0%) [0]	0 (0.0%) [0]	0 (0.0%) [0]	0 (0.0%) [0]
Infections and infestations	0 (0.0%) [0]	1 (0.4%) [1]	1 (0.4%) [2]	3 (1.2%) [3]	1 (0.4%) [1]
Conjunctivitis	0 (0.0%) [0]	0 (0.0%) [0]	1 (0.4%) [1]	0 (0.0%) [0]	0 (0.0%) [0]
Influenza	0 (0.0%) [0]	1 (0.4%) [1]	0 (0.0%) [0]	0 (0.0%) [0]	0 (0.0%) [0]
Upper respiratory tract infection	0 (0.0%) [0]	0 (0.0%) [0]	1 (0.4%) [1]	1 (0.4%) [1]	0 (0.0%) [0]
Respiratory tract infection	0 (0.0%) [0]	0 (0.0%) [0]	0 (0.0%) [0]	1 (0.4%) [1]	0 (0.0%) [0]
COVID-19	0 (0.0%) [0]	0 (0.0%) [0]	0 (0.0%) [0]	1 (0.4%) [1]	1 (0.4%) [1]

Injury, poisoning and procedural complications	0 (0.0%) [0]	0 (0.0%) [0]	0 (0.0%) [0]	0 (0.0%) [0]	0 (0.0%) [0]
Road traffic accident	0 (0.0%) [0]	0 (0.0%) [0]	0 (0.0%) [0]	0 (0.0%) [0]	0 (0.0%) [0]

Virginia Tobacco, N=260; Autumn Tobacco, N=237; Polar Menthol, N=262; Summer Menthol, N=249; Ruby Menthol, N=234.

Note. Participants reporting more than one event in a category are counted only once for that category.

*n (%) is the number and percent of participants with AEs and [E] is the total number of AEs reported, as multiple AEs could occur per session.
